# Paleotemperature Proxies from Leaf Fossils Reinterpreted in Light of Evolutionary History

**DOI:** 10.1371/journal.pone.0015161

**Published:** 2010-12-22

**Authors:** Stefan A. Little, Steven W. Kembel, Peter Wilf

**Affiliations:** 1 Department of Geosciences, Pennsylvania State University, University Park, Pennsylvania, United States of America; 2 Department of Plant Sciences, University of California Davis, Davis, California, United States of America; 3 Center for Ecology and Evolutionary Biology, University of Oregon, Eugene, Oregon, United States of America; Raymond M. Alf Museum of Paleontology, United States of America

## Abstract

Present-day correlations between leaf physiognomic traits (shape and size) and climate are widely used to estimate paleoclimate using fossil floras. For example, leaf-margin analysis estimates paleotemperature using the modern relation of mean annual temperature (MAT) and the site-proportion of untoothed-leaf species (NT). This uniformitarian approach should provide accurate paleoclimate reconstructions under the core assumption that leaf-trait variation principally results from adaptive environmental convergence, and because variation is thus largely independent of phylogeny it should be constant through geologic time. Although much research acknowledges and investigates possible pitfalls in paleoclimate estimation based on leaf physiognomy, the core assumption has never been explicitly tested in a phylogenetic comparative framework. Combining an extant dataset of 21 leaf traits and temperature with a phylogenetic hypothesis for 569 species-site pairs at 17 sites, we found varying amounts of non-random phylogenetic signal in all traits. Phylogenetic vs. standard regressions generally support prevailing ideas that leaf-traits are adaptively responding to temperature, but wider confidence intervals, and shifts in slope and intercept, indicate an overall reduced ability to predict climate precisely due to the non-random phylogenetic signal. Notably, the modern-day relation of proportion of untoothed taxa with mean annual temperature (NT-MAT), central in paleotemperature inference, was greatly modified and reduced, indicating that the modern correlation primarily results from biogeographic history. Importantly, some tooth traits, such as number of teeth, had similar or steeper slopes after taking phylogeny into account, suggesting that leaf teeth display a pattern of exaptive evolution in higher latitudes. This study shows that the assumption of convergence required for precise, quantitative temperature estimates using present-day leaf traits is not supported by empirical evidence, and thus we have very low confidence in previously published, numerical paleotemperature estimates. However, interpreting qualitative changes in paleotemperature remains warranted, given certain conditions such as stratigraphically closely-spaced samples with floristic continuity.

## Introduction

In a seminal 1915 paper, Bailey and Sinnott proposed “a botanical index of Cretaceous and Tertiary climates” [1]: in extant mesic floras, the proportion of woody “dicot” species that have untoothed leaf margins (NT) is positively related to mean annual temperature (MAT), and thus quantifying untoothed taxa in fossil floras is informative about past temperatures. Because this relationship occurs across different continents and biomes containing various plant lineages, the authors suggested that environmental convergence is the most important explanatory factor, rather than phylogenetic causes [1], [2]. Therefore, the pattern should hold through time, and paleotemperature can be inferred from fossil floras without precise systematic information, an especially useful benefit because isolated fossil leaves, the most common type of plant fossil, are notoriously difficult to identify [3], [4].

Paleobotanists have since continued to make extensive use of Bailey and Sinnott's “index,” eventually developing a quantitative method known as leaf-margin analysis, based on linear regressions of extant proportions of untoothed species (NT) and mean annual temperature (MAT) [5]–[9]. More recently, multivariate methods to quantify temperature and other climate variables have been proposed that also use NT and additional site-based means of numerous leaf-physiognomic (size and shape) variables; crucially, these methods still derive most of their predictive power for temperature from the NT-MAT correlation [8], [10]–[14]. To date, hundreds of papers have been produced regarding leaf physiognomy, climate, and paleoclimate, following the ideas of Bailey and Sinnott in a “taxon-free” approach, i.e., without phylogenetic considerations [15]; many of these are compiled in an extended topical bibliography in File S1. The general, often-stated consensus (but see below) is that the climatic distribution of leaf physiognomic traits should be similar in the past and that phylogeny is a negligible component.

In particular, precise, quantitative paleotemperature estimates from taxon-free approaches operate under a still-untested core assumption that leaf-trait variation principally results from adaptive environmental convergence, and because it is thus largely independent of phylogeny it should be constant through geologic time. If the assumption is valid, then the current uniformitarian applications are warranted, and modern trait-climate relations should estimate past climate in a quantitatively precise manner. However, certain observations cast doubt on the core assumption [2], [16]–[19]. First, leaf-climate correlations, including that of NT-MAT, vary sometimes considerably across biogeographic regions, suggesting an historical influence on trait-climate relationships [2], [9], [16], [19]–[25]. Second, many clades have obligate traits independent of the environment, including being typically toothed or untoothed (e.g., Betulaceae, Lauraceae, Myrtaceae, many *Nothofagus*, and Rosaceae), indicating phylogenetic signal [16], [26], [27]. Third, the relative richness of species with particular traits is affected by factors other than temperature, including differential origination or extinction among clades [16], [20], [26], [28], suggesting possible inconstancy of trait-climate relationships through time. Fourth, adaptations to climate change can involve many aspects of plant biology, including anatomy, physiology and biochemistry, and thus leaf traits are not necessarily expected to respond strongly to climate, depending on the nature of correlations between leaf traits and other aspects of the phenotype [29]–[32]. Despite these considerations raised in the literature, the issues above are usually considered to have insignificant effects when reconstructing paleoclimates.

An additional, more theoretical consideration is that even under an ideal model of adaptive convergence without phylogenetic signal, directional selection is expected to affect the assumption of constancy through time. Given a hypothetically unchanging climate, with constant species composition, directional selection should shift trait values over time; producing an increase in number of teeth over geological time under a constant low MAT for example. Thus, lineage persistence may contribute to some observed changes in leaf-traits through geologic time, unrelated to climate change. Further, given that lineages are expected to have differing rates of evolution, extinction and species radiation, trait-climate relations should vary through deep time, even if adaptive leaf-trait responses were ideal [26], [33].

The issues above are important because if present-day trait-climate relations do not have the expected adaptive explanation, or were not constant over evolutionary time, then the core assumption of the current uniformitarian approach would not be valid (i.e., leaf physiognomy-climate relations may have differed in the past). In this case, one would require additional information in order to precisely quantify a past trait-climate relation with confidence, such as data regarding phylogenetic placement of difficult-to-identify fossil leaves, or independent, highly accurate and well-correlated climate proxies. We note that several examples of temperature estimates from isotopic data are considered broadly concordant with associated leaf-physiognomy estimates [34]–[37]; however, these confirm qualitative temperature changes (presence of warming and cooling) much more robustly than they support precise quantitative estimates from leaf physiognomic data. The development of phylogenetic comparative methods provides a novel approach to investigate calibration floras used in paleoclimate estimates. Here, we use phylogenetic comparative methods to provide a more explicit investigation into the influence of phylogenetic history in modern-day leaf-trait variation with temperature.

We test the assumption of adaptive convergence of leaf traits with temperature by quantifying phylogenetic signal in a dataset comprising 21 leaf-physiognomic traits and MAT among 569 species-site pairs at 17 sites in the eastern USA and Barro Colorado Island, Republic of Panamá, published by Huff *et al*. [38] and Royer *et al*.[12]. The dataset shows strong correlations between leaf-physiognomic traits and MAT [12], and is ideal for a conservative test of phylogenetic effects: it is restricted to a region widely used to calibrate “leaf paleothermometers,” and it thus minimizes biogeographic effects on leaf parameters to a scale where they usually are considered unimportant [8], [10], [12]. In addition, these tests are conservative with regard to the effect of phylogenetic signal because the phylogenetic hypothesis used is resolved only to the family level (see Materials and Methods). Further, to determine the effects of evolutionary history on adaptive interpretations of leaf physiognomy–temperature relationships, we compared non-phylogenetic and phylogenetic model parameters and fits of trait-temperature relationships.

We note that the issues addressed by our analysis are separate from the numerous additional sources of uncertainty in leaf paleothermometry that have been noted, including environmental, taphonomic/preservational, sampling, and scoring biases [8], [15], [16], [33], [39]–[41].

## Methods

The Huff *et al*. and Royer *et al.* dataset [12], [38] comprises 21 leaf traits of woody “dicot” angiosperms representing 569 species-site pairs at 17 sites in the eastern USA and Barro Colorado Island, Republic of Panamá, over a range in MAT of 5.6–25.8°C. Of the 21 leaf-traits, 10 are perimeter- and area-derived measurements and 11 are tooth measurements, or ratios of tooth measurements with perimeter- and/or area-derived measurements [12], [38]. The Panamá site has the highest mean annual temperature and species richness, which includes lineages not found at the other sites, so its effects are also addressed (see below). We compared model parameters and fits of trait-MAT relationships between non-phylogenetic and phylogenetic generalized least squares (GLS and *p*GLS; [42]) to determine the effects of evolutionary history on the prevailing adaptive interpretations of leaf physiognomy–MAT relationships. Models were compared using the Akaike Information Criterion (AIC), which evaluates the fit of a model taking into account differences in the number of parameters included in the model [43]. Models with lower AIC scores are considered more parsimonious and more strongly supported by the data.

### Phylogeny construction

We created a phylogenetic hypothesis for the species included in this study by grafting them onto a family-level phylogenetic supertree of the angiosperms [44] using the Phylomatic toolkit [45]. Genera were placed as polytomies within families, and species found at several sites were placed as polytomies at the species level; each species at each site was placed as a tip of the phylogeny. Branch lengths in the resulting phylogeny represent estimates of clade age based on spacing undated nodes evenly between dated nodes in the original supertree to produce an ultrametric tree [45]. The resulting phylogeny maintained the resolution of phylogenetic relationships at the family/genus level and below, providing a conservative data set to test for the influence of phylogenetic history on traits and relationships among traits.

### Phylogenetic signal

Phylogenetic signal is a tendency for closely related taxa to possess similar trait values due to descent from a common ancestor. Phylogenetic signal in all traits, including MAT, was measured using the *K* statistic, which compares the observed phylogenetic signal in a trait to the signal expected under a Brownian motion model of evolution [46], [47]. Higher values of *K* indicate stronger phylogenetic signal (greater similarity of closely related species), with *K* = 1 expected for traits evolving under a Brownian motion model of evolution [47]. The statistical significance (*P*-values) of the *K* values for each trait was assessed by comparing the variance of independent contrasts for each trait to the expected values under a tip shuffling algorithm [47]. This *P*-value provides a test of whether the phylogenetic signal in each trait is greater than the null expectation of no signal, while the *K* statistic provides an estimate of the magnitude of phylogenetic signal. All measures of *K* were calculated using the picante R package [48]. The Panamá site's effects are addressed by measuring *K* with this site removed (Table 1).

**Table 1:** Leaf trait-MAT phylogenetic signal (*K*) and GLS model results (K statistic P-value <0.001 for all traits).

	All data		BCI removed		Nonphylogenetic model					Phylogenetic model (branch lengths scaled)					
Trait	*K*	N	*K*	N	Y-int	SE	Slope	SE	AIC	Y-int	SE	Slope	SE	λ	AIC
MAT	0.62	569	0.18	413	—	—	—	—	—	—	—	—	—	—	—
Margin untoothed (ternary)	0.51	569	0.44	413	−0.87	0.10	1.13	0.09	632.6	0.35	0.18	0.31	0.07	1.00	181.0
Margin untoothed (binomial)	—	—	—	—	−2.98	0.27	0.16	0.01	—	−0.02	0.75	0.04	0.02	—	—
Blade area	0.28	569	0.31	413	1.42	0.10	0.02	0.09	661.8	2.11	0.21	−0.38	0.09	0.94	431.4
Perimeter	0.25	569	0.28	413	1.55	0.06	−0.09	0.05	0.4	1.84	0.11	−0.27	0.05	0.91	−236.0
Internal Perimeter	0.35	325	0.34	294	1.52	0.07	−0.15	0.06	−63.5	1.78	0.10	−0.25	0.05	0.85	−270.5
Perimeter ratio	0.31	323	0.35	293	0.15	0.02	−0.08	0.02	−964.0	0.12	0.03	−0.06	0.01	0.89	−1154.5
Compactness	0.23	569	0.26	413	1.69	0.04	−0.22	0.03	−460.7	1.61	0.07	−0.19	0.04	0.77	−623.1
Shape factor	0.27	569	0.28	413	−0.59	0.04	0.21	0.03	−466.9	−0.51	0.07	0.18	0.04	0.77	−626.9
Major axis length	0.20	569	0.25	413	0.87	0.05	0.10	0.04	−132.5	1.27	0.10	−0.17	0.05	0.93	−338.4
Minor axis length	0.38	569	0.36	413	0.81	0.06	−0.14	0.05	122.3	1.10	0.13	−0.26	0.05	0.97	−173.3
Feret diameter	0.28	569	0.31	413	0.75	0.05	0.01	0.04	−94.8	1.10	0.11	−0.19	0.05	0.94	−326.0
Feret diameter ratio	0.70	569	0.39	413	−0.01	0.02	−0.08	0.01	−1287.3	−0.18	0.04	−0.01	0.01	0.99	−1573.4
Tooth area	0.75	325	0.59	294	0.66	0.18	−0.79	0.16	528.1	0.66	0.35	−0.54	0.14	0.97	368.3
Tooth area : blade area	0.45	324	0.34	293	−1.12	0.14	−0.49	0.13	373.3	−1.59	0.31	−0.02	0.11	1.00	250.7
Tooth area : perimeter	1.01	325	0.81	294	−1.07	0.14	−0.56	0.13	390.3	−1.31	0.31	−0.18	0.11	1.00	240.3
Tooth area : internal perimeter	0.92	325	0.73	294	−0.92	0.15	−0.64	0.14	429.3	−1.19	0.32	−0.24	0.11	1.00	272.8
Number of primary teeth	0.36	325	0.40	294	2.06	0.14	−0.54	0.13	398.8	1.59	0.26	−0.39	0.09	1.00	135.2
Number of secondary teeth	0.49	150	0.51	143	1.36	0.26	−0.47	0.25	227.8	1.14	0.29	−0.56	0.16	0.94	130.9
Number of teeth	0.37	325	0.43	294	2.16	0.15	−0.60	0.14	430.3	1.65	0.27	−0.43	0.10	1.00	156.6
Average tooth area	1.88	325	1.73	294	−1.49	0.20	−0.20	0.18	601.5	−1.00	0.31	−0.09	0.11	1.00	253.3
Number of teeth : perimeter	0.49	324	0.63	293	0.46	0.17	−0.38	0.15	483.6	−0.22	0.25	−0.15	0.09	1.00	120.8
Number of teeth : internal perimeter	0.52	324	0.70	293	0.60	0.17	−0.47	0.16	505.2	−0.11	0.26	−0.21	0.09	1.00	143.7

*Notes:* Compactness  =  perimeter^2^/blade area (dimensionless); Feret diameter  =  diameter of circle with same area as leaf (cm); Feret diameter ratio  =  feret diameter/major axis length (dimensionless); Shape factor  =  4π × blade area/perimeter^2^ (dimensionless); all other variables as defined in references [12], [38]; y-int  =  y-intercept; SE  =  Standard Error. The *K* statistic is a measure of relative phylogenetic signal; traits evolving under a Brownian motion model have an expected *K* value of 1 [47]. Significance values are based on comparisons of observed variance in phylogenetically independent contrast values to the values generated by 999 randomizations of taxa on the phylogeny; all *P*-values were ≤0.001. For each trait, the slope and intercept of nonphylogenetic and phylogenetic GLS models are presented with standard errors; GLS models use MAT as the independent variable. MAT and all traits except Margin untoothed were log_10_-transformed. Margin untoothed was treated as either a ternary or binomial (logit link binomial GLMM analysis) trait in the GLS analyses. Phylogenetic models used branch lengths scaled by the best-fit estimate of Pagel's λ parameter [51].

### Comparative analyses

Non-phylogenetic generalized least squares (GLS) and phylogenetic GLS (*p*GLS) regressions between climate and leaf traits were computed using the APE [49] and nlme [50] R packages. All leaf and climate traits except ‘Margin untoothed’ (see below) were log_10_-transformed prior to analysis in order to meet model assumptions of normality. For the phylogenetic GLS analyses, branch lengths were first scaled using the optimal value of Pagel's λ parameter [51] as calculated using the GEIGER R package [52]. The explanatory power of each model was evaluated using the Akaike Information Criterion (AIC) [43], which allows comparison of models with different numbers of estimated parameters. For each trait, the AIC scores of phylogenetic and non-phylogenetic GLS models were compared to determine whether the inclusion of phylogenetic information improved model fits, with lower AIC score indicating a better fit. We note that phylogenetic comparative methods cannot accommodate leaf-physiognomic means by site, as typically used in leaf-paleoclimate estimates, and thus we examined MAT correlations at the species-level.

### Margin untoothed

We performed analyses treating the ‘Margin untoothed’ trait as a binary and as a ternary variable to perform comparisons among the phylogenetic vs. non phylogenetic trait-temperature GLS models. Current physiognomic methods use the proportion of untoothed taxa at a site as a continuous variable in a least squares regression with site-climate variables. Because our analyses were based on species-level data and not site means, ‘Margin untoothed’ was defined as a ternary variable for each species at a site as follows: 1  =  all leaves untoothed, 0.5  =  both toothed and untoothed, 0  =  toothed. Leaf margin analysis and multivariate physiognomic methods treat the presence-absence of leaf teeth in this way to calculate the site means that are used as continuous values [10], [12], [13]. Because this variable is not truly continuous, we also performed GLS regressions treating ‘Margin untoothed’ as a binomial variable using the glmmPQL function in the MASS package [53] for the R statistical language and computing environment [54]. For this analysis, toothedness for each species at a site was coded as 1 (all specimens untoothed) or 0 (any specimens toothed). The binomial GLS regression found similar patterns of differences in slope and parameter uncertainty in phylogenetic versus nonphylogenetic GLS models, concordant with the non-binomial GLS analyses presented here (Table 1), where slope is greatly reduced after accounting for phylogeny.

## Results

**Figure 1:**
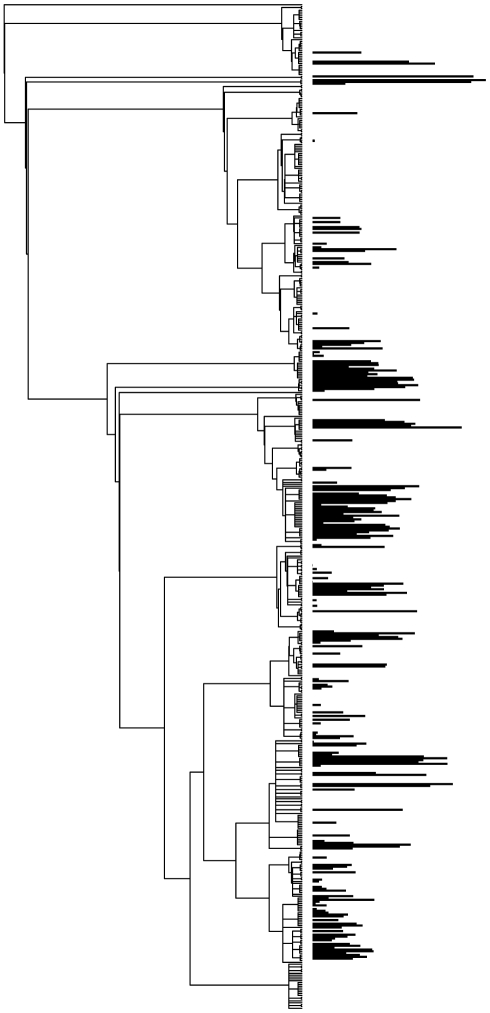
Phylogeny of all species in the community samples. Mean tooth-area character mapped at tips of the phylogeny of all species in the community samples [12] to illustrate non-random variation across the phylogeny of both presence of teeth and tooth area.

There was non-random phylogenetic signal in MAT and all measured leaf traits (Table 1: all *K, P*-values ≤0.001; Figure 1). Although all traits exhibited non-random phylogenetic signal, the amount of signal varied among traits. Tooth traits (e.g., number of teeth, average tooth area) exhibited the strongest phylogenetic signal (highest *K* values, e.g. average tooth area; *K* = 1.9). There was also phylogenetic signal in the climatic associations of taxa (*K*-value for MAT = 0.6, *P* = 0.001). Phylogenetic signal in traits and climate associations was not driven by the inclusion of the single tropical site (Barro Colorado Island, Republic of Panamá); when the tropical site was excluded (413 species-site pairs), signal remained significantly non-random for all traits and MAT (Table 1).

**Figure 2:**
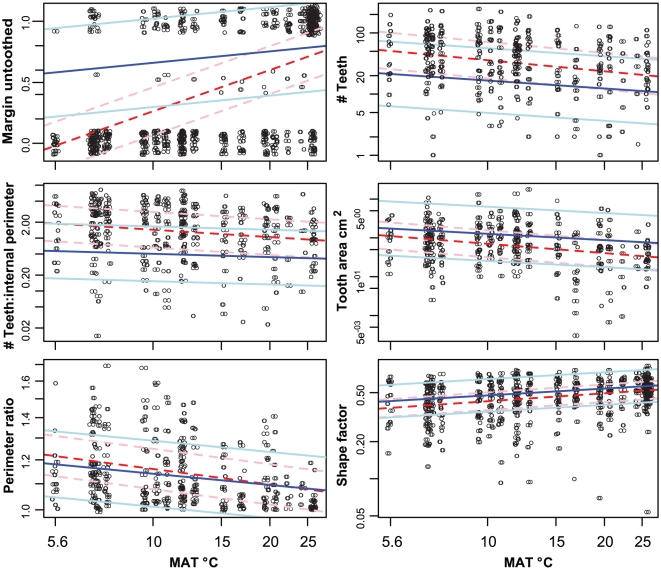
Mean annual temperature versus leaf traits. Mean annual temperature (MAT) versus the six leaf traits featured in Figure 2 of Royer *et al.* [12]. For each trait, the best-fit lines for nonphylogenetic GLS (dashed line) and phylogenetic GLS (solid line) are displayed. All traits except Margin untoothed and MAT were log_10_-transformed. Phylogenetic models used branch lengths scaled by the best-fit estimate of Pagel's λ parameter [51] (Table 1). A 95% confidence interval is displayed for each regression model (dashed rose lines  =  nonphylogenetic GLS, solid blue lines  =  phylogenetic GLS) to illustrate the increased uncertainty in predictions of climate from leaf traits when phylogeny is taken into account. Points were lightly jittered at each site to better visualize density of trait values.

All trait-climate regression models were improved by incorporation of phylogenetic relationships (Table 1; Figure 2), as demonstrated by the much lower AIC scores for phylogenetic GLS versus non-phylogenetic GLS models for all traits. The standard errors of the intercepts in *p*GLS regressions were generally higher than in non-phylogenetic GLS regressions (Table 1), leading to greater uncertainty in predictions of climate after phylogenetic relationships are taken into account.

Traits with the strongest phylogenetic signal (highest *K* values; Margin untoothed, Feret diameter ratio, Tooth area : Blade area, and Average tooth area) also showed the largest decreases in slope versus MAT after taking phylogeny into account (GLS vs. *p*GLS slopes; Figure 2, Table 1). Several trait-MAT relations with negative slopes (Perimeter, Internal Perimeter, Minor axis length, and Number of secondary teeth) or weakly positive slopes (Blade Area, Major Axis Length, and Feret Diameter) in nonphylogenetic models had steeper negative slope in *p*GLS. In general, tooth-related traits showed both strong phylogenetic signal and relatively large shifts in slopes between GLS and *p*GLS models. Conversely, non-tooth related traits such as Perimeter ratio, Compactness, and Shape Factor (a modified area:perimeter ratio), had the least altered regressions after accounting for phylogenetic relationships, in accord with their relatively low phylogenetic signal (Table 1).

## Discussion

The presence of non-random phylogenetic signal in all traits (Table 1) invalidates the core assumption that convergence dominates, and that phylogenetic history is an insignificant component of modern-day leaf-trait variation with climate. This assumption is required for “taxon-free”, quantitative leaf paleothermometry as currently used. The finding that tooth traits have the greatest amount of phylogenetic signal (highest *K* values) reinforces earlier observations that characters of leaf teeth are taxonomically informative [55]. The presence of moderate phylogenetic signal in MAT is compatible with indications of biome conservatism in plant clades through deep time [56] and supports some of the ideas behind taxon-based paleoclimate methods [17], [57]–[60]. When the tropical site was excluded, signal remained significant (Table 1), showing that phylogenetic signal among leaf traits and climate associations exists even within Eastern North America, a biogeographic region with relatively minor floristic variation that is often used for leaf-physiognomic calibrations [10], [12], [61].

The greatly improved model fits (reduced AIC values) for all *p*GLS regressions indicate that incorporation of phylogenetic information is important for understanding trait-temperature relationships (Table 1; Figure 2). Incorporating phylogenetic information into trait-climate models changed both the estimates of a relationship itself (slopes and intercepts) as well as estimates of the certainty of predictions (standard errors of slopes and intercepts). Uncertainty in prediction (standard error of intercept estimates) was increased after accounting for phylogenetic relationships, indicating that previous studies have overestimated the ability to precisely predict paleoclimate via modern-day plant traits.

The nonzero slopes of phylogenetic regressions (*p*GLS models; Table 1; Figure 2) indicate that many leaf-physiognomic traits show varying degrees of adaptive responses to temperature (as well as significant and varying phylogenetic signal as previously discussed). Importantly, this includes tooth traits other than tooth presence (e.g., number of teeth, mean tooth area), consistent with species-level observations wherein tooth traits covary with temperature across the geographic distribution of species [12], [62]. Our results are also consistent with some leaf trait responses to temperature in a single-generation experiment from seed [63] where *Acer rubrum* from a Florida population had more leaf teeth when planted in Rhode Island but still had significantly fewer teeth than the Rhode Island population, supporting the presence of both phylogenetic signal and phenotypic plasticity, as well as probable adaptive trait response in the species. Non-tooth related traits, especially Shape factor (a modified area:perimeter ratio), had the least altered correlations, in accord with their relatively low phylogenetic signal (*K*; Table 1). Recently developed tools to measure perimeter-based leaf traits show utility in detecting subtle phenotype variation among mutant plant lines [64]–[68], which suggests that other, more sensitive, perimeter- derived measurements [69], [70] could show differing values for phylogenetic signal than shown in this study.

Overall, our results support the prevailing idea that leaves are adaptively responding to climate, but that phylogenetic signal in leaf traits is responsible for a portion of variation in leaf-climate relationships, and phylogenetic information modifies our understanding of adaptive relationships between leaf physiognomic variables and climate. Several insights for improved understanding of adaptive relationships between leaf traits and temperature were revealed by our analyses; for example, Feret diameter did not appear to be strongly responsive to temperature in the dataset previously (GLS slope) [12], but after accounting for phylogenetic relationships there is a stronger relationship with temperature (i.e. steeper slope in *p*GLS vs. GLS; Table 1; Figure 2). This suggests that the influence of phylogenetic history can possibly mask leaf-trait climate relations that are not discernible in standard correlations that do not incorporate phylogenetic information.

Although we expected to find some influence of phylogenetic signal in the leaf traits and their relation to temperature, the most surprising results involved the high signal in presence of leaf teeth (Margin Untoothed). This trait is the basis of leaf-margin analysis (NT-MAT relation) and a key component of all multivariate approaches that estimate paleotemperature [8], [10]–[14], [71]. After accounting for phylogenetic relationships among species, there is an extremely weak relationship between presence of leaf teeth and temperature (NT-MAT *p*GLS; Figure 2), including an order of magnitude (in log space) decrease in slope, approaching a flat line, and substantially wider confidence intervals (Figure 2; Table 1). This pattern was observed whether presence of leaf teeth was treated as a binomial or ternary variable (Table 1). Because the NT-MAT correlation is fundamental for both univariate and multivariate paleotemperature inference as widely practiced, this result impacts most paleotemperature proxies using leaf physiognomy, as well as prevailing adaptive interpretations of leaf-teeth with temperature [7,8,10,12, S1–S351].

The relations between MAT and the traits Feret diameter ratio, Tooth area : blade area, and Average tooth area also had phylogenetic regressions with highly flattened slopes, similar to that of the NT-MAT relation (Figure 2; Table 1). Importantly, from this same data-set, Feret diameter ratio, and Tooth area : blade area were considered among the most useful adaptive traits in multivariate approaches to temperature inference based on standard trait-climate correlations [12].

The weak relationship between presence of leaf teeth and temperature after accounting for phylogeny indicates that the prevailing adaptive scenario since 1915 [1], whereby temperature is the primary force explaining evolutionary gain or loss of leaf teeth, is inaccurate. The small, but non-zero, slope of the phylogenetic regression suggests that temperature is at most only weakly related to the evolutionary gain and loss of teeth, and thus the proportion of toothed species in a flora at a given temperature would not be expected to be constant through time. The strong phylogenetic signal in leaf-tooth presence we observed is consistent with our observations of Southern Hemisphere data that clearly show phylogenetic conservatism in tooth presence or absence, including regional studies of Chilean and Australian forests [19], [72] and among the lineages compiled in a large whole-Hemisphere dataset [56]. Interestingly, as stated above, temperature does appear to have a large influence on the evolution of tooth traits other than presence of teeth (Number of teeth, Tooth area, etc; Figures 1, 2; Table 1).

Because our results indicate that historical events unrelated to temperature contributed to the majority of the present-day distribution of toothed lineages, the modern NT-MAT relationship, which is variable at a global scale, now deserves renewed investigation [73], [74]. Because both temperature and inheritance influence traits of leaf teeth (number of teeth, tooth area, etc.; Figure 2; Table 1), we suggest that a component of the modern-day distribution of untoothed species may be explained as an exaptive scenario where cool-temperature selection acted on preexisting toothed lineages (Table 1; Figure 2) [74]. Temperature selection on already-toothed lineages may have affected diversification and thereby increased the proportional richness of toothed taxa relative to warmer places and times. Thus, under this scenario, the modern-day NT-MAT correlation is primarily a result of biogeographic history, and secondarily altered by effects from exaptive temperature selection on toothed lineages.

The cool-temperature selection scenario, suggested here, is also provisionally consistent with the fossil record, in which many characteristically, or commonly, toothed clades (e.g. Betulaceae, temperate *Nothofagus* lineages, Ulmaceae, Rosaceae, Vitaceae) radiated at middle and high latitudes under warmer climates than today [59], [75]–[77]. The groups remained and further speciated at these latitudes through Cenozoic global cooling beginning in the Eocene. In this scenario, the incumbent, already toothed lineages would have been exapted and shown subsequent adaptive changes in traits of teeth (not their presence-absence) during cooling, hypothetically influencing speciation rates over time.

Observations of changing relative presence of leaf teeth through deep time that are qualitatively validated by correlation to independent temperature proxies, are often considered to be related to the migrations of clades along temperature gradients (e.g., latitude, altitude) [35]–[37], [78]–[80]. However, the same studies typically invoke convergent adaptive response to justify the application of quantitative paleotemperature estimates from the same fossil floras. Our results indicate that clade migrations along temperature gradients were probably the principal causes of the observed changes in the proportion of species with leaf teeth in these fossil floras. Inference of paleoclimate from relative proportions of toothed species will only be accurate to the extent that the distribution of toothed species and the patterns of biogeographic migration along paleotemperature gradients were the same in the past as today. As we have shown, this assumption is not warranted because of the weak adaptive relationship between relative presence of teeth and temperature, after accounting for phylogeny, which greatly decreases confidence in estimates of paleoclimate.

Qualitative analyses, i.e., detection of relative warming and cooling, remain justified using physiognomic data from well-understood regional floras that have supporting data on taxonomy, paleogeography, and distribution of traits along independently inferred paleotemperature gradients (i.e. using floras from several latitudinally adjacent basins). In practical terms, these conditions are met for several heavily studied assemblages (i.e., latest Cretaceous and Paleogene floras of the Western USA and Germany). However, we reiterate that although physiognomic *trait-latitude* gradients clearly existed at many times and places in the past with varying similarity to the modern day [33], [39], [81], the explicit *trait-temperature* gradients are usually unknown in the past. Thus, although past trait-latitude gradients allow for qualitative climate inference, confidence is very low for quantitative models of past trait-temperature gradients based directly from modern gradients as currently practiced. This point is well-demonstrated by discrepancies between taxonomic and physiognomic paleoclimate estimates, even when derived by the same workers from the same fossil floras [82], [83]. Explicit reconstruction of past gradients could potentially surmount this problem but would require major advances in independent paleotemperature proxies and their precise correlation to fossil floras.

Despite our results, it may be tempting to continue relying on current, taxon-free leaf physiognomy to generate quantitatively inferred paleotemperature estimates, by using traits that show clear adaptive responses to temperature (i.e. Number of teeth), using traits that display only slightly altered phylogenetic regressions with temperature (i.e., Shape factor), using standard leaf-margin analysis as a convenient proxy for the presence of adaptive tooth-trait response, or relying on multivariate approaches [7], [10], [12] in the hope that using many traits, or site averages of traits, will reduce prediction error. In light of our results, we advise against the above strategies because all leaf traits show non-random phylogenetic signal (Figure 1; Table 1). The use of site-averages, single-variable proxies, or multiple traits will not remove this underlying signal or reconcile the inherent uncertainties from non-independent data, and would mask the true uncertainty in prediction error. If phylogenetic signal in leaf traits is a general global phenomenon, as we strongly expect, then broader sampling of present-day “calibration” floras [84] would have the counterintuitive effect of increasing the influence of phylogenetic signal in leaf physiognomic data as more regions of the angiosperm tree of life would be sampled.

Regional differences in the relationship between proportions of toothed species and temperature (NT-MAT), wherein temperature estimates for a given value of NT differ by >5°C, may well be due to differences in phylogenetic history among biogeographic regions [9], [15], [16], [21]–[23], [25], [84], and may provide a rough empirical approximation of uncertainty due to phylogenetic history. However, the amount of extant variation may not apply to the past. For example, a large proportion of early-diverging angiosperm lineages are toothed [85] and tropical [86], suggesting possible shifting correlations through time, consistent with observations of the earliest angiosperm leaf assemblages having atypical latitudinal distributions of toothed leaves [39].

In summary, we have demonstrated that there is evidence for an adaptive response to temperature in many leaf traits. However, the presence of non-random phylogenetic signal throughout leaf physiognomic data leads to leaf trait-climate relationships that are driven both by adaptive evolution and phylogenetic history, and the adaptive signal is especially weak for the most widely used variable, presence of teeth. Non-independence of species data due to phylogenetic relatedness results in conventional, non-phylogenetic models of leaf trait-climate relationships underestimating the true uncertainty in estimates of paleotemperature from leaf traits. An approach that should permit reliable qualitative estimation of change in paleoclimate from leaf traits would be to use leaf physiognomic variables that show the strongest evolutionary correlations with climate based on *p*GLS models. These variables should be able to detect relative temperature changes through time, especially over geologically short intervals with floristic continuity, given sufficient evidence that the trait-climate relationships were not greatly altered in the past.

## Supporting Information

File S1 (DOCX)
