# Supplementary material for: Paleotemperature Proxies from Leaf Fossils Reinterpreted in Light of Evolutionary History
Source: PLoS One. 2010 Dec 22;5(12):e15161. doi: 10.1371/journal.pone.0015161 (PMC3008682; doi:10.1371/journal.pone.0015161)
Supplement: File S1 — (DOCX) [file pone.0015161.s001.docx]

**Supporting Information**

We provide, in reverse chronological order, an extended topical bibliography [S1–S351] to demonstrate the historic and continuing importance of the correlations of leaf physiognomy and temperature. Most of the cited literature is directly affected by this paper because it either features, or uses extant vegetation to calibrate and interpret, quantitative and qualitative paleotemperature estimates derived from fossil leaf physiognomy. In many cases numerous estimates, each from a corresponding fossil flora, are given in a single paper. However, we also include a large selection of the most influential related papers, especially those that address living and fossil leaf-teeth, often but not always in a climatic context. These include critiques of the paleotemperature approach; taphonomic work on leaf-margin preservation; historic observations and ecological studies of leaf teeth; paleoclimate modeling results that take paleobotanical temperature estimates into account as constraints; morphometric, phylogenetic, and developmental-genetic studies of leaf toothing and lobing; and physiological and modeling studies of water, carbon, and heat flux in leaf teeth. We exclude purely descriptive work and the abundant literature that considers leaf size but not margin features. This bibliography is available electronically by request to P. Wilf.

Extended Bibliography

S1. Peppe DJ, Royer DL, Cariglino B, Oliver SY, Newman S, Leight E, Enikolopov G, Fernandez-Burgos M, Herrera F, Adams JM, Correa E, Currano ED, Erickson JM, Hinojosa LF, Iglesias A, Jaramillo CA, Johnson KR, Jordan GJ, Kraft N, Lovelock EC, Lusk CH, Niinemets Ü, Peñuelas J, Rapson G, Wing SL, Wright IJ (in press) Sensitivity of leaf size and shape to climate: global patterns and paleoclimatic applications. New Phytol.

S2. Albarán-Lara AL, Mendoza-Cuenca L, Valencia-Avalos S, González-Rodríguez A, Oyama K (2010) Leaf fluctuating asymmetry increases with hybridization and introgression between *Quercus magnoliifolia* and *Quercus resinosa* (Fagaceae) through an altitudinal gradient in Mexico. Int J Plant Sci 171: 310-322.

S3. Archibald SB, Bossert WH, Greenwood DR, Farrell BD (2010) Seasonality, the latitudinal gradient of diversity, and Eocene insects. Paleobiology 36: 374-398.

S4. Backhaus A, Kuwabara A, Bauch M, Monk N, Sanguinetti G, Fleming A (2010) LEAFPROCESSOR: a new leaf phenotyping tool using contour bending energy and shape cluster analysis. New Phytol 187: 251-261.

S5. Currano ED, Labandeira CC, Wilf P (2010) Fossil insect folivory tracks paleotemperature for six million years. Ecol Monogr 80: 547-567.

S6. Herman AB, Spicer RA (2010) Mid-Cretaceous floras and climate of the Russian high Arctic (Novosibirsk Islands, Northern Yakutiya). Palaeogeogr Palaeoclimatol Palaeoecol 295: 409-422.

S7. Hinojosa LF, Pérez F, Gaxiola A, Sandoval I (2010) Historical and phylogenetic constraints on the incidence of entire leaf margins: insights from a new South American model. Global Ecol Biogeogr:DOI: 10.1111/j.1466-8238.2010.00595.x.

S8. Lazarević Z, Milivojević J (2010) Early Miocene flora of the intramontane Žagubica Basin (Serbian Carpatho-Balkanides). Neues Jahrbuch für Geologie und Paläontologie-Abhandlungen 256: 141-150.

S9. Peppe DJ, Royer DL, Wilf P, Kowalski EA (2010) Quantification of large uncertainties in fossil leaf paleoaltimetry. Tectonics 29: TC3015, doi:10.1029/2009TC002549.

S10. Pole M (2010) Ecology of Paleocene-Eoecene vegetation at Kakahu, South Canterbury, New Zealand. Palaeontol Electron 13: article 14A.

S11. Pole M, Philippe M (2010) Cretaceous plant fossils of Pitt Island, the Chatham group, New Zealand. Alcheringa 34: 231-263.

S12. Smith RY, Greenwood DR, Basinger JF (2010) Estimating paleoatmospheric *p*CO_2_ during the Early Eocene Climatic Optimum from stomatal frequency of *Ginkgo*, Okanagan Highlands, British Columbia, Canada. Palaeogeogr Palaeoclimatol Palaeoecol 293: 120-131.

S13. Spicer RA, Herman AB (2010) The Late Cretaceous environment of the Arctic: a quantitative reassessment based on plant fossils. Palaeogeogr Palaeoclimatol Palaeoecol 295: 423-442.

S14. Su T, Xing Y-W, Liu Y-S, Jacques FMB, Chen W-Y, Huang Y-J, Zhou Z-K (2010) Leaf margin analysis: a new equation from humid to mesic forests in China. Palaios 25: 234-238.

S15. Chew AE (2009) Paleoecology of the early Eocene Willwood mammal fauna from the central Bighorn Basin, Wyoming. Paleobiology 35: 13-31.

S16. Dilcher DL, Kowalski EA, Wiemann MC, Hinojosa LF, Lott TA (2009) A climatic and taxonomic comparison between leaf litter and standing vegetation from a Florida swamp woodland. Am J Bot 96: 1108-1115.

S17. Head JJ, Bloch JI, Hastings AK, Bourque JR, Cadena EA, Herrera FA, Polly PD, Jaramillo CA (2009) Giant boid snake from the Palaeocene neotropics reveals hotter past equatorial temperatures. Nature 457: 715-717.

S18. Herman AB, Akhmetiev MA, Kodrul TM, Moiseeva MG, Iakovleva AI (2009) Flora development in northeastern Asia and northern Alaska during the Cretaceous-Paleogene transitional epoch. Stratigr Geol Correl 17: 79-97.

S19. Herman AB (2009) Paleobotany and the Earth's climate: looking into the future from the geological past (in Russian). Vestn Ross Akad Nauk 79: 387-393.

S20. Jones CS, Bakker FT, Schlichting CD, Nicotra AB (2009) Leaf shape evolution in the South African genus *Pelargonium* L' Hér. (Geraniaceae). Evolution 63: 479-497.

S21. Malhado ACM, Whittaker RJ, Malhi Y, Ladle RJ, Ter Steege H, Butt N, Aragão LEOC, Quesada CA, Murakami-Araujo A, Phillips OL, Peacock J, López-González G, Baker TR, Anderson LO, Arroyo L, Almeida S, Higuchi N, Killeen TJ, Monteagudo A, Neill DA, Pitman NCA, Prieto A, Salamão RP, squez-M. R, Laurance WF, Ramírez H (2009) Spatial distribution and functional significance of leaf lamina shape in Amazonian forest trees. Biogeosciences 6: 1577-1590.

S22. Royer DL, Kooyman RM, Little SA, Wilf P (2009) Ecology of leaf teeth: a multi-site analysis from eastern Australian subtropical rainforest. Am J Bot 96: 738-750.

S23. Royer DL, Meyerson LA, Robertson KM, Adams JM (2009) Phenotypic plasticity of leaf shape along a temperature gradient in *Acer rubrum*. PLoS One 4: e7653.

S24. Smith RY, Basinger JF, Greenwood DR (2009) Depositional setting, fossil flora, and paleoenvironment of the Early Eocene Falkland site, Okanagan Highlands, British Columbia. Can J Earth Sci 46: 811-822.

S25. Spicer RA, Valdes PJ, Spicer TEV, Craggs HJ, Srivastava G, Mehrotra RC, Yang J (2009) New developments in CLAMP: Calibration using global gridded meteorological data. Palaeogeogr Palaeoclimatol Palaeoecol 283: 91-98.

S26. Steart DC, Greenwood DR, Boon PI (2009) The chemical constraints upon leaf decay rates: taphonomic implications among leaf species in Australian terrestrial and aquatic environments. Rev Palaeobot Palynol 157: 358-374.

S27. Su T, Xing Y-W, Yang Q-S, Zhou Z-K (2009) Reconstruction of mean annual temperature in Chinese Eocene paleofloras based on leaf margin analysis. Acta Palaeontologica Sinica 48: 65-72.

S28. Tamás J, Hably L (2009) Unidirectional shift in leaf morphology of coexisting species -- a possible indicator of palaeoclimatic differences. Palaeogeogr Palaeoclimatol Palaeoecol 271: 185-195.

S29. Thrasher BL, Sloan LC (2009) Carbon dioxide and the early Eocene climate of western North America. Geology 37: 807-810.

S30. Váchova Z, Kvaček J (2009) Palaeoclimate analysis of the flora of the Klikov Formation, Upper Cretaceous, Czech Republic. Bull Geosci 84: 257-268.

S31. Wing SL, Herrera F, Jaramillo CA, Gómez-N. C, Wilf P, Labandeira CC (2009) Late Paleocene fossils from the Cerrejón Formation, Colombia, are the earliest record of Neotropical rainforest. Proc Natl Acad Sci U S A 106: 18627-18632.

S32. Wong, N. H. and Chen, Y. (2009) Tropical urban heat islands. New York: Taylor and Francis. 264 p.

S33. Woodburne MO, Gunnell GF, Stucky RK (2009) Climate directly influences Eocene mammal faunal dynamics in North America. Proc Natl Acad Sci U S A 106: 13399-13403.

S34. Woodburne.M.O., Gunnell GF, Stucky RK (2009) Land mammal faunas of North America rise and fall during the early Eocene climatic optimum. Denver Museum of Nature & Science Annals 1: 1-78.

S35. Woodcock D, Meyer H, Dunbar N, McIntosh W, Prado I, Morales G (2009) Geologic and taphonomic context of El Bosque Petrificado Piedra Chamana (Cajamarca, Peru). Geol Soc Am Bull 121: 1172-1178.

S36. Wu J, Sun B, Liu Y-S, Xie S, Lin Z (2009) A new species of *Exbucklandia* (Hamamelidaceae) from the Pliocene of China and its paleoclimatic significance. Rev Palaeobot Palynol 155: 32-41.

S37. Xia K, Su T, Liu Y-S, Xing Y-W, Jacques FMB, Zhou Z (2009) Quantitative climate reconstructions of the late Miocene Xiaolongtan megaflora from Yunnan, southwest China. Palaeogeogr Palaeoclimatol Palaeoecol 276: 80-86.

S38. Xu F, Guo W, Xu W, Wei Y, Wang R (2009) Leaf morphology correlates with water and light availability: what consequences for simple and compound leaves? Progress in Natural Science 19: 1789-1798.

S39. Adams JM, Green WA, Zhang Y (2008) Leaf margins and temperature in the North American flora: recalibrating the paleoclimatic thermometer. Global Planet Change 60: 523-534.

S40. Aizen MA, Ezcurra C (2008) Do leaf margins of the temperate forest flora of southern South America reflect a warmer past? Global Ecol Biogeogr 17: 164-174.

S41. Blein T, Pulido A, Vialette-Guiraud A, Nikovics K, Morin H, Hay A, Johansen IE, Tsiantis M, Laufs P (2008) A conserved molecular framework for compound leaf development. Science 322: 1835-1839.

S42. Bylesjö M, Segura V, Soolanayakanahally RY, Rae AM, Trygg J, Gustafsson P, Jansson S, Street NR (2008) LAMINA: a tool for rapid quantification of leaf size and shape parameters. BMC Plant Biol 8:82: doi:10.1186/1471-2229-8-82.

S43. Currano ED, Wilf P, Wing SL, Labandeira CC, Lovelock EC, Royer DL (2008) Sharply increased insect herbivory during the Paleocene-Eocene thermal maximum. Proc Natl Acad Sci U S A 105: 1960-1964.

S44. DiMichele WA, Gastaldo RA (2008) Plant paleoecology in deep time. Ann Mo Bot Gard 95: 144-198.

S45. Francis JE, Ashworth A, Cantrill DJ, Crame JA, Howe J, et al. (2008) 100 Million Years of Antarctic climate evolution: evidence from fossil plants. In: Cooper AK, Barrett PJ, Stagg H, Storey B, Stump E et al., editors. Antarctica: a keystone in a changing world. Washington, DC: National Academies Press. pp. 19-27.

S46. Kvaček Z (2008) Tertiary vegetation of Europe and its dynamics and climatic signal -- new approaches in botany of the past. Palaeobotanist 57: 459-463.

S47. Prasad M, Pandey SM (2008) Plant diversity and climate during Siwalik (Miocene-Pliocene) in the Himalayan foot hills of western Nepal. Palaeontogr Abt B 278: 13-70.

S48. Royer DL, McElwain JC, Adams JM, Wilf P (2008) Sensitivity of leaf size and shape to climate within *Acer rubrum* and *Quercus kelloggii*. New Phytol 179: 808-817.

S49. Spicer RA, Ahlberg A, Herman AB, Hofmann C-C, Raikevich M, Valdes PJ, Markwick PJ (2008) The Late Cretaceous continental interior of Siberia: a challenge for climate models. Earth Planet Sci Lett 267: 228-235.

S50. Uribe-Salas D, Sáenz-Romero C, González-Rodríguez A, Téllez-Valdéz O, Oyama K (2008) Foliar morphological variation in the white oak *Quercus rugosa* Née (Fagaceae) along a latitudinal gradient in Mexico: potential implications for management and conservation. Forest Ecol Manage 256: 2121-2126.

S51. Wilf P (2008) Fossil angiosperm leaves: paleobotany's difficult children prove themselves. Paleontol Soc Pap 14: 319-333.

S52. Wilf P (2008) Insect-damaged fossil leaves record food web response to ancient climate change and extinction. New Phytol 178: 486-502.

S53. Xu F, Guo W, Xu W, Wang R (2008) Habitat effects on leaf morphological plasticity in *Quercus acutissima*. Acta Biologica Cracoviensia 50: 19-26.

S54. Yabe A (2008) Early Miocene terrestrial climate inferred from plant megafossil assemblages of the Joban and Soma areas, Northeast Honshu, Japan. Bull Geol Surv Jpn 59: 397-413.

S55. Bogner J, Johnson KR, Kvaček Z, Upchurch GR, Jr. (2007) New fossil leaves of Araceae from the Late Cretaceous and Paleogene of western North America. Zitteliana 47: 133-147.

S56. Buechler WK, Dunn MT, Rember WC (2007) Late Miocene Pickett Creek flora of Owyhee County, Idaho. Contrib Mus Paleontol Univ Mich 31: 305-362.

S57. Christophel DC, Gordon PJ (2007) Foliar physiognomy and taphonomy as tools for understanding the paleoclimate of Australia's fossil rainforests. Cour Forschungsinst Senckenb 258: 159-169.

S58. Dos-Santos MA, Bernardes-de-Oliveira ME, Sant'Anna LG (2007) Evidências paleoclimáticas e paleoecológicas, segundo dados paleobotânicos e mineralógicos, dos argilitos Neógenos de Jaguariúna (SP), correlatos à Formação Rio Claro. Rev UNG - Geoci 6: 80-106.

S59. Doyle JA (2007) Systematic value and evolution of leaf architecture across the angiosperms in light of molecular phylogenetic analyses. Cour Forschungsinst Senckenb 258: 21-37.

S60. Dutra TL (2007) Paleobotany and paleoclimatology. Part II: leaf assemblages (taphonomy, paleoclimatology and paleogeography). In: Koutsoukos EAM, editors. Applied stratigraphy. Dordrecht, Netherlands: Springer. pp. 194-202.

S61. Erwin DM, Schick KN (2007) New Miocene oak galls (Cynipini) and their bearing on the history of cynipid wasps in western North America. J Paleontol 81: 568-580.

S62. Forest CE (2007) Paleoaltimetry: a review of thermodynamic methods. Rev Mineral Geochem 66: 173-193.

S63. Greenwood DR (2007) Fossil angiosperm leaves and climate: from Wolfe and Dilcher to Burnham and Wilf. Cour Forschungsinst Senckenb 258: 95-108.

S64. Herman AB, Kvaček J (2007) Early Campanian Grünbach flora of Austria: systematic composition and palaeoclimatic interpretations. Acta Palaeobot 47: 37-55.

S65. Iglesias A, Wilf P, Johnson KR, Zamuner AB, Cúneo NR, Matheos SD, Singer BS (2007) A Paleocene lowland macroflora from Patagonia reveals significantly greater richness than North American analogs. Geology 35: 947-950.

S66. Krieger JD, Guralnick RP, Smith DM (2007) Generating empirically determined, continuous measures of leaf shape for paleoclimate reconstruction. Palaios 22: 212-219.

S67. Kvaček Z, Teodoridis V (2007) Tertiary macrofloras of the Bohemian Massif: a review with correlations within Boreal and Central Europe. Bull Geosci 82: 383-408.

S68. Kvaček Z (2007) Do extant nearest relatives of thermophile European Cenozoic plant elements reliably reflect climatic signal? Palaeogeogr Palaeoclimatol Palaeoecol 253: 32-40.

S69. Liu Y-J, Arens NC, Li C-S (2007) Range change in *Metasequoia*: relationship to palaeoclimate. Bot J Linn Soc 154: 115-127.

S70. Martinetto E, Uhl D, Tarabra E (2007) Leaf physiognomic indications for a moist warm-temperate climate in NW Italy during the Messinian (Late Miocene). Palaeogeogr Palaeoclimatol Palaeoecol 253: 41-55.

S71. Meyer HW (2007) A review of paleotemperature - lapse rate methods for estimating paleoelevation from fossil floras. Rev Mineral Geochem 66: 155-171.

S72. Spicer RA (2007) Recent and future developments of CLAMP: building on the legacy of Jack A. Wolfe. Cour Forschungsinst Senckenb 258: 109-118.

S73. Traiser C, Uhl D, Mosbrugger V (2007) Leaf physiognomy and palaeoenvironmental estimates: an alternative technique based on an European calibration. Acta Palaeobot 47: 183-201.

S74. Uhl D, Traiser C, Griesser U, Denk T (2007) Fossil leaves as palaeoclimate proxies in the Palaeogene of Spitsbergen (Svalbard). Acta Palaeobot 47: 89-107.

S75. Uhl D, Klotz S, Traiser C, Thiel C, Utescher T, Kowalski E, Dilcher DL (2007) Cenozoic paleotemperatures and leaf physiognomy -- a European perspective. Palaeogeogr Palaeoclimatol Palaeoecol 248: 24-31.

S76. Upchurch GR, Jr., Lomax BH, Beerling DJ (2007) Paleobotanical evidence for climatic change across the Cretaceous-Tertiary boundary, North America: twenty years after Wolfe and Upchurch. Cour Forschungsinst Senckenb 258: 57-74.

S77. Yang J, Wang Y-F, Spicer RA, Mosbrugger V, Li C-S, Sun Q-G (2007) Climatic reconstruction at the Miocene Shanwang basin, China, using leaf margin analysis, CLAMP, coexistence approach, and overlapping distribution analysis. Am J Bot 94: 599-608.

S78. Zidianakis G, Mohr BAR, Fassoulas C (2007) A late Miocene leaf assemblage from Vrysses, western Crete, Greece, and its paleoenvironmental and paleoclimatic interpretation. Geodiversitas 29: 351-377.

S79. Bortiri E, Vanden Heuvel B, Potter D (2006) Phylogenetic analysis of morphology in *Prunus* reveals extensive homoplasy. Pl Syst Evol 259: 53-71.

S80. Carpenter RJ, Hill RS, Scriven LJ (2006) Palmately lobed Proteaceae leaf fossils from the Middle Eocene of South Australia. Int J Plant Sci 167: 1049-1060.

S81. Green WA (2006) Loosening the CLAMP: an exploratory graphical approach to the Climate Leaf Analysis Multivariate Program. Palaeontol Electron 9: art. 9A.

S82. Harris N (2006) The elevation history of the Tibetan Plateau and its implications for the Asian monsoon. Palaeogeogr Palaeoclimatol Palaeoecol 241: 4-15.

S83. Hayes PA, Francis JE, Cantrill DJ, Crame JA (2006) Paleoclimate analysis of Late Cretaceous angiosperm leaf floras, James Ross Island, Antarctica. Geol Soc London Spec Publ 258: 49-62.

S84. Hinojosa LF, Pesce O, Yabe A, Uemura K, Nishida H (2006) Physiognomical analysis and paleoclimate of the Ligorio Márquez Formation, central Patagonia, XI Region, Chile. In: Nishida H, editors. Post-Cretaceous floristic changes in Southern Patagonia, Chile. Tokyo: Faculty of Science and Engineering, Chuo University. pp. 45-55.

S85. Hinojosa LF, Armesto JJ, Villagrán C (2006) Are Chilean coastal forests pre-Pleistocene relicts? Evidence from foliar physiognomy, palaeoclimate, and phytogeography. J Biogeogr 33: 331-341.

S86. McMillan ME, Heller PL, Wing SL (2006) History and causes of post-Laramide relief in the Rocky Mountain orogenic plateau. Geol Soc Am Bull 118: 393-405.

S87. Miller IM, Brandon MT, Hickey LJ (2006) Using leaf margin analysis to estimate the mid-Cretaceous (Albian) paleolatitude of the Baja BC block. Earth Planet Sci Lett 245: 95-114.

S88. Neto JC, Meyer GE, Jones DD, Samal AK (2006) Plant species identification using Elliptic Fourier leaf shape analysis. Comput Electron Agric 50: 121-134.

S89. Royer DL, Wilf P (2006) Why do toothed leaves correlate with cold climates? Gas exchange at leaf margins provides new insights into a classic paleotemperature proxy. Int J Plant Sci 167: 11-18.

S90. Sewall JO, Sloan LC (2006) Come a little bit closer: a high-resolution climate study of the early Paleogene Laramide foreland. Geology 34: 81-84.

S91. Teodoridis V, Kvaček Z (2006) Palaeobotanical research of the early Miocene deposits overlying the main coal seam (Libkovice and Lom members) in the Most Basin (Czech Republic). Bull Geosci 81: 93-113.

S92. Uhl D, Bruch AA, Traiser C, Klotz S (2006) Palaeoclimate estimates for the middle Miocene Schrotzburg flora (S Germany): a multi-method approach. Int J Earth Sci 95: 1071-1085.

S93. Wilf P, Labandeira CC, Johnson KR, Ellis B (2006) Decoupled plant and insect diversity after the end-Cretaceous extinction. Science 313: 1112-1115.

S94. Burnham RJ, Ellis B, Johnson KR (2005) Modern tropical forest taphonomy: does high biodiversity affect paleoclimatic interpretations? Palaios 20: 439-451.

S95. Craggs HJ (2005) Late Cretaceous climate signal of the Northern Pekulney Range flora of northeastern Russia. Palaeogeogr Palaeoclimatol Palaeoecol 217: 25-46.

S96. Dillhoff RM, Leopold EB, Manchester SR (2005) The McAbee flora of British Columbia and its relation to the early-middle Eocene Okanagan Highlands flora of the Pacific Northwest. Can J Earth Sci 42: 151-166.

S97. Feild TS, Sage TL, Czerniak C, Iles WJD (2005) Hydathodal leaf teeth of *Chloranthus japonicus* (Chloranthaceae) prevent guttation-induced flooding of the mesophyll. Plant Cell Environ 28: 1179-1190.

S98. Gayó E, Hinojosa LF, Villagrán C (2005) On the persistence of tropical paleofloras in central Chile during the early Eocene. Rev Palaeobot Palynol 137: 41-50.

S99. Greenwood DR (2005) Leaf margin analysis: taphonomic constraints. Palaios 20: 498-505.

S100. Greenwood DR (2005) Leaf form and the reconstruction of past climates. New Phytol 166: 355-357.

S101. Greenwood DR, Archibald SB, Mathewes RW, Moss PT (2005) Fossil biotas from the Okanagan Highlands, southern British Columbia and northeastern Washington State: climates and ecosystems across an Eocene landscape. Can J Earth Sci 42: 167-185.

S102. Hinojosa LF (2005) Cambios climáticos y vegetacionales inferidos a partir de paleofloras Cenozoicas del sur de Sudamérica. Rev Geol Chil 32: 95-115.

S103. Hinojosa LF, Villagrán C (2005) Did South American Mixed Paleofloras evolve under thermal equability or in the absence of an effective Andean barrier during the Cenozoic? Palaeogeogr Palaeoclimatol Palaeoecol 217: 1-23.

S104. Moiseeva MG (2005) Members of the angiosperm genus *Cissites* from the Maastrichtian of the Koryak flora (northeastern Russia). Paleontol J 39: 548-556.

S105. Poole I, Cantrill D, Utescher T (2005) A multi-proxy approach to determine Antarctic terrestrial palaeoclimate during the Late Cretaceous and Early Tertiary. Palaeogeogr Palaeoclimatol Palaeoecol 222: 95-121.

S106. Quan C, Zhang L (2005) An analysis of the early Paleogene climate of the Jiayin area, Heilongjiang province. Dizhi Lunping (Geol Rev ) 51: 10-15.

S107. Royer DL, Wilf P, Janesko DA, Kowalski EA, Dilcher DL (2005) Correlations of climate and plant ecology to leaf size and shape: potential proxies for the fossil record. Am J Bot 92: 1141-1151.

S108. Spicer RA, Herman AB, Kennedy EM (2005) The sensitivity of CLAMP to taphonomic loss of foliar physiognomic characters. Palaios 20: 429-438.

S109. Traiser C, Klotz S, Uhl D, Mosbrugger V (2005) Environmental signals from leaves — a physiognomic analysis of European vegetation. New Phytol 166: 465-484.

S110. Wilf P, Johnson KR, Cúneo NR, Smith ME, Singer BS, Gandolfo MA (2005) Eocene plant diversity at Laguna del Hunco and Río Pichileufú, Patagonia, Argentina. Am Nat 165: 634-650.

S111. Wing SL, Harrington GJ, Smith FA, Bloch JI, Boyer DM, Freeman KH (2005) Transient floral change and rapid global warming at the Paleocene-Eocene boundary. Science 310: 993-996.

S112. Burnham RJ, Johnson KR (2004) South American palaeobotany and the origins of neotropical rainforests. Phil Trans R Soc London Ser B 359: 1595-1610.

S113. Fricke HC, Wing SL (2004) Oxygen isotope and paleobotanical estimates of temperature and δ^18^O-latitude gradients over North America during the early Eocene. Am J Sci 304: 612-635.

S114. Glasspool IJ, Hilton J, Collinson ME, Wang SJ, Li CS (2004) Foliar physiognomy in Cathaysian gigantopterids and the potential to track Palaeozoic climates using an extinct plant group. Palaeogeogr Palaeoclimatol Palaeoecol 205: 69-110.

S115. Greenwood DR, Wilf P, Wing SL, Christophel DC (2004) Paleotemperature estimation using leaf-margin analysis: is Australia different? Palaios 19: 129-142.

S116. Herman AB (2004) Quantitative paleobotanical data: constraints on Late Cretaceous climates in Eurasia and Alaska. In: Leonov IG, editors. Klimat v epokhi krupnykh biosfernykh perestroek (Climates in the epochs of major biospheric transformations). Moscow: Nauka. pp. 88-104.

S117. Jacobs BF, Herendeen PS (2004) Eocene dry climate and woodland vegetation in tropical Africa reconstructed from fossil leaves from northern Tanzania. Palaeogeogr Palaeoclimatol Palaeoecol 213: 115-123.

S118. Kvaček Z, Walther H (2004) Oligocene flora of Bechlejovice near Decin from the neovolcanic area of the Ceske Stredohori mountains, Czech Republic. Acta Mus Natl Pragae, Ser B, Nat Hist 60: 9-60.

S119. Retallack GJ, Orr WN, Prothero DR, Duncan RA, Kester PR, Ambers CP (2004) Eocene-Oligocene extinction and paleoclimatic change near Eugene, Oregon. Geol Soc Am Bull 116: 817-839.

S120. Roth-Nebelsick A, Utescher T, Mosbrugger V, Diester-Haass L, Walther H (2004) Changes in atmospheric CO_2_ concentrations and climate from the late Eocene to early Miocene: palaeobotanical reconstruction based on fossil floras from Saxony, Germany. Palaeogeogr Palaeoclimatol Palaeoecol 205: 43-67.

S121. Spicer RA, Herman AB, Kennedy EM (2004) Foliar physiognomic record of climatic conditions during dormancy: Climate Leaf Analysis Multivariate Program (CLAMP) and the cold month mean temperature. J Geol 112: 685-702.

S122. Wilf P, Johnson KR (2004) Land plant extinction at the end of the Cretaceous: a quantitative analysis of the North Dakota megafloral record. Paleobiology 30: 347-368.

S123. Barclay RS, Johnson KR, Betterton WJ, Dilcher DL (2003) Stratigraphy and megaflora of a K-T boundary section in the eastern Denver Basin, Colorado. Rocky Mount Geol 38: 45-71.

S124. Ellis B, Johnson KR, Dunn RE (2003) Evidence for an *in situ* early Paleocene rainforest from Castle Rock, Colorado. Rocky Mount Geol 38: 73-100.

S125. Gomez B, Barale G, Saad D, Perrichot V (2003) Santonian angiosperm-dominated leaf-assemblage from Piolenc (Vaucluse, Sud-Est de la France). C R Palevol 2: 197-204.

S126. Greenwood DR, Moss PT, Rowett AI, Vadala AJ, Keefe RL (2003) Plant communities and climate change in southeastern Australia during the early Paleogene. Geol Soc Am Spec Pap 369: 365-380.

S127. Huff PM, Wilf P, Azumah EJ (2003) Digital future for paleoclimate estimation from fossil leaves? Preliminary results. Palaios 18: 266-274.

S128. Hunt RJ, Poole I (2003) Paleogene West Antarctic climate and vegetation history in light of new data from King George Island. Geol Soc Am Spec Pap 369: 395-412.

S129. Johnson KR, Reynolds ML, Werth KW, Thomasson JR (2003) Overview of the Late Cretaceous, early Paleocene, and early Eocene megafloras of the Denver Basin, Colorado. Rocky Mount Geol 38: 101-120.

S130. Kennedy EM (2003) Late Cretaceous and Paleocene terrestrial climates of New Zealand: leaf fossil evidence from South Island assemblages. N Z J Geol Geophys 46: 295-306.

S131. Kowalski EA, Dilcher DL (2003) Warmer paleotemperatures for terrestrial ecosystems. Proc Natl Acad Sci U S A 100: 167-170.

S132. Liang M-M, Bruch A, Collinson M, Mosbrugger V, Li C-S, Sun Q-G, Hilton J (2003) Testing the climatic estimates from different palaeobotanical methods: an example from the middle Miocene Shanwang flora of China. Palaeogeogr Palaeoclimatol Palaeoecol 198: 279-301.

S133. Myers JA (2003) Terrestrial Eocene-Oligocene vegetation and climate of the Pacific Northwest. In: Prothero DR, Ivany LC, Nesbitt E, editors. From greenhouse to icehouse: the marine Eocene-Oligocene transition. New York: Columbia University Press. pp. 171-185.

S134. Schneider JV, Zipp D, Gaviria J, Zizka G (2003) Successional and mature stands in an upper Andean rain forest transect of Venezuela: do leaf characteristics of woody species differ? J Trop Ecol 19: 251-259.

S135. Spicer RA, Harris NBW, Widdowson M, Herman AB, Guo S, Valdes PJ, Wolfe JA, Kelley SP (2003) Constant elevation of southern Tibet over the past 15 million years. Nature 421: 622-624.

S136. Uhl D, Mosbrugger V, Bruch A, Utescher T (2003) Reconstructing palaeotemperatures using leaf floras - case studies for a comparison of leaf margin analysis and the coexistence approach. Rev Palaeobot Palynol 126: 49-64.

S137. Wilf P, Johnson KR, Huber BT (2003) Correlated terrestrial and marine evidence for global climate changes before mass extinction at the Cretaceous-Paleogene boundary. Proc Natl Acad Sci U S A 100: 599-604.

S138. Wilf P, Cúneo NR, Johnson KR, Hicks JF, Wing SL, Obradovich JD (2003) High plant diversity in Eocene South America: evidence from Patagonia. Science 300: 122-125.

S139. Aguilar FJ, Velasco de León P (2002) El clima durante el Plioceno en la región de Santa María Amajac, Hidalgo, México. Bol Soc Bot México 71: 71-81.

S140. Gregory-Wodzicki KM (2002) A late Miocene subtropical-dry flora from the northern Altiplano, Bolivia. Palaeogeogr Palaeoclimatol Palaeoecol 180: 331-348.

S141. Herman AB, Kvaček J (2002) Campanian Grünbach flora of Lower Austria: preliminary floristics and palaeoclimatology. Ann Naturhist Mus Wien 103A: 1-21.

S142. Herman AB, Spicer RA, Kvaček J (2002) Late Cretaceous climate of Eurasia and Alaska: a quantitative palaeobotanical approach. In: Wagreich M, editors. Aspects of Cretaceous stratigraphy and palaeobiogeography. Österrische Akademie der Wissenschaften 15. pp. 93-108.

S143. Jacobs BF (2002) Estimation of low-latitude paleoclimates using fossil angiosperm leaves: examples from the Miocene Tugen Hills, Kenya. Paleobiology 28: 399-421.

S144. Johnson KR, Ellis B (2002) A tropical rainforest in Colorado 1.4 million years after the Cretaceous-Tertiary boundary. Science 296: 2379-2383.

S145. Kennedy EM, Spicer RA, Rees PM (2002) Quantitative palaeoclimate estimates from Late Cretaceous and Paleocene leaf floras in the northwest of the South Island, New Zealand. Palaeogeogr Palaeoclimatol Palaeoecol 184: 321-345.

S146. Kowalski EA (2002) Mean annual temperature estimation based on leaf morphology: a test from tropical South America. Palaeogeogr Palaeoclimatol Palaeoecol 188: 141-165.

S147. MacLeod N (2002) Geometric morphometrics and geological shape-classification systems. Earth-Science Reviews 59: 27-47.

S148. Spicer RA, Ahlberg A, Herman AB, Kelley SP, Raikevich MI, Rees PM (2002) Palaeoenvironment and ecology of the middle Cretaceous Grebenka flora of northeastern Asia. Palaeogeogr Palaeoclimatol Palaeoecol 184: 65-105.

S149. Sun Q-G, Collinson ME, Li C-S, Wang Y-F, Beerling DJ (2002) Quantitative reconstruction of palaeoclimate from the middle Miocene Shanwang flora, eastern China. Palaeogeogr Palaeoclimatol Palaeoecol 180: 315-329.

S150. Troncoso A, Suárez M, De la Cruz R, Palma-Heldt S (2002) Paleoflora de la Formación Ligorio Márquez (XI Región, Chile) en su localidad tipo: sistemática, edad e implicancias

paleoclimáticas. Rev Geol Chil 29: 113-135.

S151. Velázquez-Rosas N, Meave J, Vázquez-Santana S (2002) Elevational variation of leaf traits in montane rain forest tree species at La Chinantla, Southern México. Biotropica 34: 534-546.

S152. Burnham RJ, Pitman NCA, Johnson KR, Wilf P (2001) Habitat-related error in estimating temperatures from leaf margins in a humid tropical forest. Am J Bot 88: 1096-1102.

S153. Graham A, Gregory-Wodzicki KM, Wright KL (2001) Studies in Neotropical paleobotany. XV. A Mio-Pliocene palynoflora from the Eastern Cordillera, Bolivia: implications for the uplift history of the Central Andes. Am J Bot 88: 1545-1557.

S154. Leopold EB, Clay-Poole ST (2001) Florissant leaf and pollen floras of Colorado compared: Climatic implications. In: Evanoff E, Gregory-Wodzicki KM, Johnson KR, editors. Fossil flora and Stratigraphy of the Florissant Formation, Colorado. Denver, Colorado: Denver Museum of Nature & Science. pp. 17-70.

S155. Meyer HW (2001) A review of paleoelevation estimates from the Florissant flora, Colorado. In: Evanoff E, Gregory-Wodzicki KM, Johnson KR, editors. Fossil flora and stratigraphy of the Florissant Formation, Colorado. Denver, Colorado: Denver Museum of Nature & Science. pp. 205-216.

S156. Wiemann MC, Dilcher DL, Manchester SR (2001) Estimation of mean annual temperature from leaf and wood physiognomy. Forest Sci 47: 141-149.

S157. Wing SL, Harrington GJ (2001) Floral response to rapid warming in the earliest Eocene and implications for concurrent faunal change. Paleobiology 27: 539-563.

S158. Doyle JA, Endress PK (2000) Morphological phylogenetic analysis of basal angiosperms: comparison and combination with molecular data. Int J Plant Sci 161: S121-S153.

S159. Golovneva LB (2000) Palaeogene climates of Spitsbergen. GFF 122: 62-63.

S160. Golovneva LB (2000) The Maastrichtian (Late Cretaceous) climate in the Northern Hemisphere. Geol Soc London Spec Publ 181: 43-54.

S161. Gregory-Wodzicki KM (2000) Relationships between leaf morphology and climate, Bolivia: implications for estimating paleoclimate from fossil floras. Paleobiology 26: 668-688.

S162. Gregory-Wodzicki KM (2000) Uplift history of the Central and Northern Andes: A review. Geol Soc Am Bull 112: 1091-1105.

S163. Groot EP, Meicenheimer RD (2000) Comparison of leaf plastochron index and allometric analyses of tooth development in *Arabidopsis thaliana*. J Plant Growth Regul 19: 77-89.

S164. Kvaček Z (2000) Climatic oscillations versus environmental changes in the interpretation of Tertiary plant assemblages. Geol Soc London Spec Publ 181: 89-94.

S165. Sewall JO, Sloan LC, Huber M, Wing SL (2000) Climate sensitivity to changes in land surface characteristics. Global Planet Change 26: 445-465.

S166. Spicer RA (2000) Leaf physiognomy and climate change. In: Culver SJ, Rawson P, editors. Biotic response to global change: the last 145 million years. Cambridge: Cambridge University Press. pp. 244-264.

S167. Utescher T, Mosbrugger V, Ashraf AR (2000) Terrestrial climate evolution in northwest Germany over the last 25 million years. Palaios 15: 430-449.

S168. Wilf P (2000) Late Paleocene-early Eocene climate changes in southwestern Wyoming: paleobotanical analysis. Geol Soc Am Bull 112: 292-307.

S169. Wing SL, Bao H, Koch PL (2000) An early Eocene cool period? Evidence for continental cooling during the warmest part of the Cenozoic. In: Huber BT, MacLeod K, Wing SL, editors. Warm climates in earth history. Cambridge: Cambridge University Press. pp. 197-237.

S170. Wing SL (2000) Cool, warm, cool, warm - climate oscillation and floral change during the Paleocene/Eocene boundary interval. GFF 122: 182-183.

S171. Wolfe JA, Dilcher DL (2000) Late Paleocene through middle Eocene climates in lowland North America. GFF 122: 184-185.

S172. Wolfe JA, Gregory-Wodzicki KM, Molnar P, Mustoe G (2000) Paleobotanical evidence for the development of high altitudes during the early Eocene in northwestern North America. GFF 122: 186-187.

S173. Barrón E (1999) Estudio paleobotánico del afloramiento Vallesiense (Neógeno) del Barranco de Salanca (La Cerdaña, Lérida, España). Aspectos paleoecológicos. An Jard Bot Madrid 57: 81-96.

S174. Forest CE, Wolfe JA, Molnar P, Emanuel KA (1999) Paleoaltimetry incorporating atmospheric physics and botanical estimates of paleoclimate. Geol Soc Am Bull 111: 497-511.

S175. Herendeen PS, Magallón-Puebla S, Lupia R, Crane PR, Kobylinska J (1999) A preliminary conspectus of the Allon flora from the Late Cretaceous (late Santonian) of Central Georgia, U.S.A. Ann Mo Bot Gard 86: 407-471.

S176. Jacobs BF (1999) Estimation of rainfall variables from leaf characters in tropical Africa. Palaeogeogr Palaeoclimatol Palaeoecol 145: 231-250.

S177. Kelley SP, Spicer RA, Herman AB (1999) New ^40^Ar/^39^Ar dates for Cretaceous Chauna Group tephra, north-eastern Russia, and their implications for the geologic history and floral evolution of the North Pacific region. Cretaceous Res 20: 97-106.

S178. McIver EE, Basinger JF (1999) Early Tertiary floral evolution in the Canadian high Arctic. Ann Mo Bot Gard 86: 523-545.

S179. Upchurch GR, Jr., Otto-Bliesner BL, Scotese CR (1999) Terrestrial vegetation and its effects on climate during the latest Cretaceous. Geol Soc Am Spec Pap 332: 407-426.

S180. Wilf P, Wing SL, Greenwood DR, Greenwood CL (1999) Using fossil leaves as paleoprecipitation indicators: an Eocene example: Reply. Geology 27: 92.

S181. Wilf P, Labandeira CC (1999) Response of plant-insect associations to Paleocene-Eocene warming. Science 284: 2153-2156.

S182. Wolfe JA, Spicer RA (1999) Fossil leaf character states: multivariate analyses. In: Jones TP, Rowe NP, editors. Fossil plants and spores: modern techniques. London: Geological Society of London. pp. 233-239.

S183. Chase CG, Gregory-Wodzicki KM, Parrish JT, DeCelles PG (1998) Topographic history of the Western Cordillera of North America and controls on climate. In: Crowley TJ, Burke KC, editors. Tectonic boundary conditions for climate reconstructions. New York: Oxford University Press. pp. 73-99.

S184. Ferguson DK, Pingen M, Zetter R, Hofmann C-C (1998) Advances in our knowledge of the Miocene plant assemblage from Kreuzau, Germany. Rev Palaeobot Palynol 101: 147-177.

S185. Gandolfo MA, Marenssi SA, Santillana SN (1998) Flora y paleoclima de la Formación La Meseta (Eoceno medio), Isla Marambio (Seymour), Antártida. Asoc Paleontol Argent Publ Espec 5: 155-162.

S186. Gregory-Wodzicki KM, McIntosh WC, Velasquez K (1998) Climatic and tectonic implications of the late Miocene Jakokkota flora, Bolivian Altiplano. J S Am Earth Sci 11: 533-560.

S187. Hably L, Kvaček Z (1998) Pliocene mesophytic forests surrounding crater lakes in western Hungary. Rev Palaeobot Palynol 101: 257-269.

S188. McLellan T, Endler JA (1998) The relative success of some methods for measuring and describing the shape of complex objects. Syst Biol 47: 264-281.

S189. Moraczewski IR (1998) Analyzing leaf margins with the use of a shape feature description language. Can J Bot 76: 552-560.

S190. Parrish, J. T. (1998) Interpreting pre-Quaternary climate from the geologic record. New York: Columbia University Press. 338 p.

S191. Parrish JT, Daniel IL, Kennedy EM, Spicer RA (1998) Paleoclimatic significance of mid-Cretaceous floras from the middle Clarence Valley, New Zealand. Palaios 13: 149-159.

S192. Sloan LC, Morrill C (1998) Orbital forcing and Eocene continental temperatures. Palaeogeogr Palaeoclimatol Palaeoecol 144: 21-35.

S193. Smith GA, Manchester SR, Ashwill M, McIntosh WC, Conrey RM (1998) Late Eocene-early Oligocene tectonism, volcanism, and floristic change near Gray Butte, central Oregon. Geol Soc Am Bull 110: 759-778.

S194. Spicer RE, Herman AB (1998) Cretaceous climate of Asia and Alaska: a comparison of paleobotanical evidence with a climate computer model. Paleontol J 32: 105-118.

S195. Wiemann MC, Manchester SR, Dilcher DL, Hinojosa LF, Wheeler EA (1998) Estimation of temperature and precipitation from morphological characters of dicotyledonous leaves. Am J Bot 85: 1796-1802.

S196. Wilf P, Beard KC, Davies-Vollum KS, Norejko JW (1998) Portrait of a late Paleocene (early Clarkforkian) terrestrial ecosystem: Big Multi Quarry and associated strata, Washakie Basin, southwestern Wyoming. Palaios 13: 514-532.

S197. Wilf P, Wing SL, Greenwood DR, Greenwood CL (1998) Using fossil leaves as paleoprecipitation indicators: an Eocene example. Geology 26: 203-206.

S198. Wing SL (1998) Tertiary vegetation of North America as a context for mammalian evolution. In: Janis CM, Scott KM, Jacobs LL, editors. Evolution of Tertiary mammals in North America, volume 1: terrestrial carnivores, ungulates, and ungulate-like mammals. Cambridge: Cambridge University Press. pp. 37-65.

S199. Wing SL (1998) Late Paleocene-early Eocene floral and climatic change in the Bighorn Basin, Wyoming. In: Aubry M-P, Lucas S, Berggren WA, editors. Late Paleocene-Early Eocene climatic and biotic events in the marine and terrestrial records. New York: Columbia University Press. pp. 380-400.

S200. Wolfe JA, Forest CE, Molnar P (1998) Paleobotanical evidence of Eocene and Oligocene paleoaltitudes in midlatitude western North America. Geol Soc Am Bull 110: 664-678.

S201. Baker-Brosh KF, Peet RK (1997) The ecological significance of lobed and toothed leaves in temperate forest trees. Ecology 78: 1250-1255.

S202. Burnham RJ (1997) Stand characteristics and leaf litter composition of a dry forest hectare in Santa Rosa National Park, Costa Rica. Biotropica 29: 384-395.

S203. Davies-Vollum KS (1997) Early Palaeocene palaeoclimatic inferences from fossil floras of the western interior, USA. Palaeogeogr Palaeoclimatol Palaeoecol 136: 145-164.

S204. Gregory-Wodzicki KM (1997) The late Eocene House Range flora, Sevier Desert, Utah: paleoclimate and paleoelevation. Palaios 12: 552-567.

S205. Herman AB, Spicer RA (1997) New quantitative paleoclimate data for the Late Cretaceous Arctic: evidence for a warm polar ocean. Palaeogeogr Palaeoclimatol Palaeoecol 128: 227-251.

S206. Hinojosa LF, Villagrán C (1997) Historia de los bosques del sur de Sudamérica, I: antecedentes paleobotánicos, geológicos y climáticos del Terciario del cono sur de América. Rev Chil Hist Nat 70: 225-239.

S207. Jordan GJ (1997) Uncertainty in paleoclimatic reconstructions based on leaf physiognomy. Aust J Bot 45: 527-547.

S208. Meyer HW, Manchester SR (1997) The Oligocene Bridge Creek flora of the John Day Formation, Oregon. Univ Calif Publ Geol Sci 141: 1-195.

S209. Mosbrugger V, Utescher T (1997) The coexistence approach -— a method for quantitative reconstructions of Tertiary terrestrial palaeoclimate data using plant fossils. Palaeogeogr Palaeoclimatol Palaeoecol 134: 61-86.

S210. Mustoe GE, Gannaway WL (1997) Paleogeography and paleontology of the early Tertiary Chuckanut Formation, Northwest Washington. Wash Geol 25: 3-18.

S211. Stranks L, England P (1997) The use of a resemblance function in the measurement of climatic parameters from the physiognomy of woody dicotyledons. Palaeogeogr Palaeoclimatol Palaeoecol 131: 15-28.

S212. Wilf P (1997) When are leaves good thermometers? A new case for Leaf Margin Analysis. Paleobiology 23: 373-390.

S213. Wolfe JA (1997) Relations of environmental change to angiosperm evolution during the Late Cretaceous and Tertiary. In: Iwatsuki K, Raven PH, editors. Evolution and diversification of land plants. Tokyo: Springer-Verlag. pp. 269-290.

S214. Wolfe JA, Schorn HE, Forest CE, Molnar P (1997) Paleobotanical evidence for high altitudes in Nevada during the Miocene. Science 276: 1672-1675.

S215. Greenwood DR (1996) Eocene monsoon forests in central Australia? Aust Syst Bot 9: 95-112.

S216. Gregory KM (1996) Are paleoclimate estimates biased by foliar physiognomic responses to increased atmospheric CO2? Palaeogeogr Palaeoclimatol Palaeoecol 124: 39-51.

S217. Gregory KM, McIntosh WC (1996) Paleoclimate and paleoelevation of the Oligocene Pitch-Pinnacle flora, Sawatch range, Colorado. Geol Soc Am Bull 108: 545-561.

S218. Halloy SRP, Mark AF (1996) Comparative leaf morphology spectra of plant communities in New Zealand, the Andes and the European Alps. J R Soc N Z 26: 41-78.

S219. Herman AB, Spicer RA (1996) Palaeobotanical evidence for a warm Cretaceous Arctic Ocean. Nature 380: 330-333.

S220. Jacobs BF, Deino AL (1996) Test of climate-leaf physiognomy regression models, their application to two Miocene floras from Kenya, and ^40^Ar-^39^Ar dating of the late Miocene Kapturo site. Palaeogeogr Palaeoclimatol Palaeoecol 123: 259-271.

S221. Jordan GJ (1996) Eocene continental climates and latitudinal temperature gradients: Comment. Geology 24: 1054.

S222. Kappelle M, Leal ME (1996) Changes in leaf morphology and foliar nutrient status along a successional gradient in a Costa Rican upper montane *Quercus* forest. Biotropica 28: 331-344.

S223. Kovach WL, Spicer RA (1996) Canonical Correspondence Analysis of leaf physiognomy: a contribution to the development of a new palaeoclimatological tool. Palaeoclimates 1: 125-138.

S224. Millar CI (1996) Tertiary vegetation history. In: Sierra Nevada ecosystem project: final report to Congress, vol. II, assessments and scientific basis for management options. Davis: University of California, Centers for Water and Wildland Resources. pp. 71-122.

S225. Richards, P. W. (1996) The tropical rain forest. Cambridge: Cambridge University Press. 575 p.

S226. Wing SL, Greenwood DR (1996) Eocene continental climates and latitudinal temperature gradients: Reply. Geology 24: 1054-1055.

S227. Askin RA, Spicer RA (1995) The Late Cretaceous and Cenozoic history of vegetation and climate at northern and southern high latitudes: a comparison. In: National Research Council Panel on Effects of Past Global Change on Life, editors. Effects of past global change on life. Washington, DC: National Academy of Sciences. pp. 156-173.

S228. Baghai NL, Jorstad RB (1995) Paleontology, paleoclimatology and paleoecology of the late middle Miocene Musselshell Creek flora, Clearwater County Idaho. A preliminary study of a new fossil flora. Palaios 10: 424-436.

S229. Christophel DC (1995) The impact of climatic changes on the development of the Australian flora. In: National Research Council Panel on Effects of Past Global Change on Life, editors. Effects of past global change on life. Washington, DC: National Academy of Sciences. pp. 174-183.

S230. Forest CE, Molnar P, Emanuel KA (1995) Palaeoaltimetry from energy conservation principles. Nature 374: 347-350.

S231. Greenwood DR, Wing SL (1995) Eocene continental climates and latitudinal temperature gradients. Geology 23: 1044-1048.

S232. Roth A, Mosbrugger V, Belz G, Neugebauer HJ (1995) Hydrodynamic modelling study of angiosperm leaf venation types. Bot Acta 108: 121-126.

S233. Wing SL, Alroy J, Hickey LJ (1995) Plant and mammal diversity in the Paleocene to early Eocene of the Bighorn Basin. Palaeogeogr Palaeoclimatol Palaeoecol 115: 117-155.

S234. Wolfe JA (1995) Paleoclimatic estimates from Tertiary leaf assemblages. Annu Rev Earth Plan Sci 23: 119-142.

S235. Boyd A (1994) Some limitations in using leaf physiognomic data as a precise method for determining paleoclimates with an example from the Late Cretaceous Pautût Flora of West Greenland. Palaeogeogr Palaeoclimatol Palaeoecol 112: 261-278.

S236. Burnham RJ (1994) Paleoecological and floristic heterogeneity in the plant-fossil record: an analysis based on the Eocene of Washington. U S Geol Surv Bull 2085-B: 1-36.

S237. Carpenter RJ, Hill RS, Jordan GJ (1994) Cenozoic vegetation in Tasmania: macrofossil evidence. In: Hill RS, editors. History of the Australian vegetation: Cretaceous to Recent. Cambridge: Cambridge University Press. pp. 276-298.

S238. Greenwood DR (1994) Palaeobotanical estimates for Tertiary climates. In: Hill RS, editors. History of the Australian vegetation: Cretaceous to Recent. Cambridge: Cambridge University Press. pp. 44-59.

S239. Gregory KM (1994) Palaeoclimate and palaeoelevation of the 35 Ma Florissant flora, Front Range, Colorado. Palaeoclimates 1: 23-57.

S240. Herman AB (1994) A review of Late Cretaceous floras and climates of Arctic Russia. In: Boulter MC, Fisher HC, editors. Cenozoic plants and climates of the Arctic. Berlin: Springer-Verlag. pp. 127-149.

S241. Pole M (1994) An Eocene macroflora from the Taratu Formation at Livingstone, North Otago, New-Zealand. Aust J Bot 42: 341-367.

S242. Povey DAR, Spicer RA, England PC (1994) Paleobotanical Investigation of early Tertiary palaeoelevations in northeastern Nevada: initial results. Rev Palaeobot Palynol 81: 1-10.

S243. Wolfe JA (1994) Alaskan Palaeogene climates as inferred from the CLAMP database. In: Boulter MC, Fisher HC, editors. Cenozoic plants and climates of the Arctic. Springer-Verlag. pp. 223-237.

S244. Wolfe JA (1994) Tertiary climatic changes at middle latitudes of western North America. Palaeogeogr Palaeoclimatol Palaeoecol 108: 195-205.

S245. Wolfe JA (1994) An analysis of Neogene climates in Beringia. Palaeogeogr Palaeoclimatol Palaeoecol 108: 207-216.

S246. Greenwood DR, Basinger JF (1993) Stratigraphy and floristics of Eocene swamp forests from Axel-Heiberg Island, Canadian Arctic Archipelago. Can J Earth Sci 30: 1914-1923.

S247. Herman AB (1993) Stages and cycles in the Late Cretaceous floral changes of the Anadyr'-Koryak Subregion (Northeast Russia) and their connection with climatic changes. Stratigr Geol Correl 1: 77-87.

S248. McIver EE, Basinger JF (1993) Flora of the Ravenscrag Formation (Paleocene), southwestern Saskatchewan, Canada. Palaeontogr Can 10: 1-167.

S249. McLellan T (1993) The roles of heterochrony and heteroblasty in the diversification of leaf shapes in *Begonia dregei* (Begoniaceae). Am J Bot 80: 796-804.

S250. Pole MS (1993) Early Miocene flora of the Manuherikia Group, New Zealand.10. Paleoecology and stratigraphy. J R Soc N Z 23: 393-426.

S251. Schuepp PH (1993) Leaf boundary layers. New Phytol 125: 477-507.

S252. Spicer RA, Rees PM, Chapman JL (1993) Cretaceous phytogeography and climate signals. Philos Trans R Soc London Ser B 341: 277-285.

S253. Upchurch GR, Jr., Wolfe JA (1993) Cretaceous vegetation of the Western Interior and adjacent regions of North America. Geol Assoc Can Spec Pap 39: 243-281.

S254. Wijninga VM, Kuhry P (1993) Late Pliocene paleoecology of the Guasca Valley (Cordillera Oriental, Colombia). Rev Palaeobot Palynol 78: 69-127.

S255. Wing SL, Greenwood DR (1993) Fossils and fossil climate: the case for equable continental interiors in the Eocene. Philos Trans R Soc London Ser B 341: 243-252.

S256. Wolfe JA (1993) A method of obtaining climatic parameters from leaf assemblages. U S Geol Surv Bull 2040: 1-71.

S257. Greenwood DR (1992) Taphonomic constraints on foliar physiognomic interpretations of Late Cretaceous and Tertiary paleoclimates. Rev Palaeobot Palynol 71: 149-190.

S258. Gregory KM, Chase CG (1992) Tectonic significance of paleobotanically estimated climate and altitude of the late Eocene erosion surface, Colorado. Geology 20: 581-585.

S259. Li HM (1992) Early Tertiary palaeoclimate of King George Island, Antarctica- evidence from the Fossil Hill Flora. In: Yoshida Y, editors. Recent progress in Antarctic earth science. Tokyo: Terra Scientific Publishing Company (Terrapus). pp. 371-375.

S260. Meyer HW (1992) Lapse rates and other variables applied to estimating paleoaltitudes from fossil floras. Palaeogeogr Palaeoclimatol Palaeoecol 99: 71-99.

S261. Sloan LC, Barron EJ (1992) A comparison of Eocene climate model results to quantified paleoclimatic interpretations. Palaeogeogr Palaeoclimatol Palaeoecol 93: 183-202.

S262. Wolfe JA (1992) An analysis of present-day terrestrial lapse rates in the western conterminous United States and their significance to paleoaltitudinal estimates. U S Geol Surv Bull 1964: 1-35.

S263. Wolfe JA (1992) Climatic, floristic, and vegetational changes near the Eocene/Oligocene boundary in North America. In: Prothero DR, Berggren WA, editors. Eocene-Oligocene climatic and biotic evolution. Princeton, New Jersey: Princeton University Press. pp. 421-436.

S264. Woodcock DW (1992) Climate reconstruction based on biological indicators. Q Rev Biol 67: 457-477.

S265. Wilson TP, Canny MJ, McCully ME (1991) Leaf teeth, transpiration and the retrieval of apoplastic solutes in balsam poplar. Physiol Plant 83: 225-232.

S266. Wing SL, Bown TM, Obradovich JD (1991) Early Eocene biotic and climatic change in interior western North America. Geology 19: 1189-1192.

S267. Boyd A (1990) The Thyra Ø flora: toward an understanding of the climate and vegetation during the early Tertiary in the high Arctic. Rev Palaeobot Palynol 62: 189-203.

S268. Canny MJ (1990) What becomes of the transpiration stream? New Phytol 114: 341-368.

S269. Chaloner WG, Creeber GT (1990) Do fossil plants give a climatic signal? J Geol Soc London 147: 343-350.

S270. Dolph GE (1990) A critique of the theoretical basis of leaf margin analysis. Proc Indiana Acad Sci 99: 1-10.

S271. Johnson KR, Hickey LJ (1990) Megafloral change across the Cretaceous/Tertiary boundary in the northern Great Plains and Rocky Mountains, U.S.A. Geol Soc Am Spec Pap 247: 433-444.

S272. Spicer RA, Parrish JT (1990) Late Cretaceous-early Tertiary paleoclimates of northern high latitudes: a quantitative view. J Geol Soc London 147: 329-341.

S273. Wolfe JA (1990) Palaeobotanical evidence for a marked temperature increase following the Cretaceous/Tertiary boundary. Nature 343: 153-156.

S274. Burnham RJ (1989) Relationships between standing vegetation and leaf litter in a paratropical forest: implications for paleobotany. Rev Palaeobot Palynol 58: 5-32.

S275. Christophel DC, Greenwood DR (1989) Changes in climate and vegetation in Australia during the Tertiary. Rev Palaeobot Palynol 58: 95-109.

S276. Chen J-M, Ibbetson A, Milford JR (1988) Boundary-layer resistances of artificial leaves in turbulent air II: leaves inclined to the mean flow. Bound -Lay Meteorol 45: 371-390.

S277. Parrish JT, Spicer RA (1988) Late Cretaceous terrestrial vegetation: a near-polar temperature curve. Geology 16: 22-25.

S278. White RJ, Prentice HC, Verwijst T (1988) Automated image acquisition and morphometric description. Can J Bot 66: 450-459.

S279. Givnish TJ (1987) Comparative studies of leaf form: assessing the relative roles of selective pressures and phylogenetic constraints. New Phytol 106: 131-160.

S280. Jacobs BF, Kabuye CHS (1987) A middle Miocene (12.2 my old) forest in the East African Rift Valley, Kenya. J Hum Evol 16: 147-155.

S281. Upchurch GR, Wolfe JA (1987) Mid-Cretaceous to Early Tertiary vegetation and climate: evidence from fossil leaves and woods. In: Friis EM, Chaloner WG, Crane PR, editors. The origins of Angiosperms and their biological consequences. Cambridge: Cambridge University Press. pp. 75-105.

S282. Wolfe JA, Upchurch GR, Jr. (1987) Leaf assemblages across the Cretaceous-Tertiary boundary in the Raton Basin, New Mexico and Colorado. Proc Natl Acad Sci U S A 84: 5096-5100.

S283. Wolfe JA (1987) Late Cretaceous-Cenozoic history of deciduousness and the terminal Cretaceous event. Paleobiology 13: 215-226.

S284. Wolfe JA, Upchurch GR, Jr. (1987) North American nonmarine climates and vegetation during the Late Cretaceous. Palaeogeogr Palaeoclimatol Palaeoecol 61: 33-77.

S285. Romero EJ (1986) Paleogene phytogeography and climatology of South America. Ann Mo Bot Gard 73: 449-461.

S286. Spicer RA, Parrish JT (1986) Paleobotanical evidence for cool north polar climates in middle Cretaceous (Albian-Cenomanian) time. Geology 14: 703-706.

S287. Vlcek J, Cheung E (1986) Fractal analysis of leaf shapes. Can J For Res 16: 124-127.

S288. Wolfe JA, Upchurch GR, Jr. (1986) Vegetation, climatic and floral changes at the Cretaceous-Tertiary boundary. Nature 324: 148-152.

S289. Kincaid DT, Schneider RB (1983) Quantification of leaf shape with a microcomputer and Fourier Transform. Can J Bot 61: 2333-2342.

S290. Gottschlich DE, Smith AP (1982) Convective heat-transfer characteristics of toothed leaves. Oecologia 53: 418-420.

S291. Wolfe JA, Poore RZ (1982) Tertiary marine and nonmarine climatic trends. In: Berger W, Crowell JC, editors. Climate in Earth history. Washington, DC: National Academy of Sciences. pp. 154-15.

S292. Goble-Garratt EM, Bell DT, Loneragan WA (1981) Floristic and leaf structure patterns along a shallow elevational gradient. Aust J Bot 29: 329-347.

S293. Wolfe JA (1981) Paleoclimatic significance of the Oligocene and Neogene floras of the northwestern United States. In: Niklas KJ, editors. Paleobotany, Paleoecology, and Evolution. New York: Praeger. pp. 79-101.

S294. Davis JM, Taylor SE (1980) Leaf physiognomy and climate: a multivariate analysis. Quat Res 14: 337-348.

S295. Hickey LJ (1980) Paleocene stratigraphy and flora of the Clark's Fork Basin. Univ Mich Pap Paleontol 24: 33-49.

S296. Wolfe JA (1980) Tertiary climates and floristic relationships at high latitudes in the Northern Hemisphere. Palaeogeogr Palaeoclimatol Palaeoecol 30: 313-323.

S297. Dolph G, Dilcher DL (1979) Foliar physiognomy as an aid in determining paleoclimate. Palaeontogr Abt B 170: 151-172.

S298. Dolph GE (1979) Variation in leaf margin with respect to climate in Costa Rica. Bull Torrey Bot Club 106: 104-109.

S299. Givnish TJ (1979) On the adaptive significance of leaf form. In: Solbrig OT, Jain S, Johnson GB, Raven PH, editors. Topics in plant population biology. New York: Columbia University Press. pp. 375-407.

S300. Wolfe JA (1979) Temperature parameters of humid to mesic forests of Eastern Asia and relation to forests of other regions of the Northern Hemisphere and Australasia. U S Geol Surv Prof Pap 1106: 1-37.

S301. Christophel DC, Blackburn DT (1978) Tertiary megafossil flora of Maslin Bay, South Australia: a preliminary report. Alcheringa 2: 311-319.

S302. Dudley EC (1978) Adaptive radiation in the Melastomataceae along an altitudinal gradient in Peru. Biotropica 10: 134-143.

S303. Givnish TJ (1978) Ecological aspects of plant morphology: leaf form in relation to environment. Acta Biotheor 27: 83-142.

S304. Roth JL, Dilcher DL (1978) Some considerations in leaf size and leaf margin analysis of fossil leaves. Cour Forschungsinst Senckenb 30: 165-171.

S305. Wolfe JA (1978) Paleobotanical interpretation of Tertiary climates in the Northern Hemisphere. Am Sci 66: 694-703.

S306. Hickey LJ (1977) Stratigraphy and paleobotany of the Golden Valley Formation (Early Tertiary) of western North Dakota. Geol Soc Am Mem 150: 1-183.

S307. Wolfe JA (1977) Paleogene floras from the Gulf of Alaska region. U S Geol Surv Prof Pap 997: 1-108.

S308. Hickey LJ, Wolfe JA (1975) The bases of angiosperm phylogeny: vegetative morphology. Ann Mo Bot Gard 62: 538-589.

S309. Krassilov VA (1975) Climatic changes in eastern Asia as indicated by fossil floras. II. Late Cretaceous and Danian. Palaeogeogr Palaeoclimatol Palaeoecol 17: 157-172.

S310. Leigh EG, Jr. (1975) Structure and climate in tropical rain forest. Annu Rev Ecol Syst 6: 67-86.

S311. MacGinitie HD (1974) An early middle Eocene flora from the Yellowstone-Absaroka volcanic province, northwestern Wind River Basin, Wyoming. Univ Calif Publ Geol Sci 108: 1-103.

S312. Dilcher DL (1973) A paleoclimatic interpretation of the Eocene floras of southeastern North America. In: Graham A, editors. Vegetation and vegetational history of northern Latin America. Amsterdam: Elsevier. pp. 39-59.

S313. Meyer H (1973) The Oligocene Lyons flora of northwestern Oregon. Ore Bin 35: 37-53.

S314. Volkheimer W (1971) Aspectos paleoclimatológicos del Terciario Argentino. Rev Mus Argent Ci Nat Secc Paleontol 1: 241-264.

S315. Wolfe JA (1971) Tertiary climatic fluctuations and methods of analysis of Tertiary floras. Palaeogeogr Palaeoclimatol Palaeoecol 9: 27-57.

S316. Vogel S (1970) Convective cooling at low airspeeds and the shapes of broad leaves. J Exp Bot 21: 91-101.

S317. Axelrod DI, Bailey HP (1969) Paleotemperature analysis of Tertiary floras. Palaeogeogr Palaeoclimatol Palaeoecol 6: 163-195.

S318. Baker DN, Myhre DL (1969) Effects of leaf shape and boundary layer thickness on photosynthesis in cotton (*Gossypium hirsutum*). Physiol Plant 22: 1043-1049.

S319. Gentry AH (1969) A comparison of some leaf characteristics of tropical dry forest and tropical wet forest in Costa Rica. Turrialba 19: 419-428.

S320. Howard RA (1969) The ecology of an elfin forest in Puerto Rico, 8. Studies of stem growth and form and of leaf structure. J Arnold Arbor 50: 225-261.

S321. MacGinitie HD (1969) The Eocene Green River flora of northwestern Colorado and northeastern Utah. Univ Calif Publ Geol Sci 83: 1-140.

S322. Wolfe JA, Hopkins DM (1967) Climatic changes recorded by Tertiary land floras in northwestern North America. In: Hatai K, editors. Tertiary correlations and climatic changes in the Pacific. Sendai, Japan: Sasaki. pp. 67-76.

S323. Schwarzbach M (1966) Das Klima des rheinischen Tertiärs. Z Dtsch Geol Ges 118: 33-68.

S324. Smiley CJ (1966) Cretaceous floras from Kuk River Area, Alaska: stratigraphic and climatic interpretations. Geol Soc Am Bull 77: 1-14.

S325. Wolfe JA (1966) Tertiary plants from the Cook Inlet Region, Alaska. U S Geol Surv Prof Pap 398-B: 1-32.

S326. Becker HF (1961) Paleobotanical record of solar change. Ann N Y Acad Sci 95: 684-687.

S327. Wolfe JA, Barghoorn ES (1960) Generic change in Tertiary floras in relation to age. Am J Sci 258-A: 388-399.

S328. Dorf E (1959) Climatic changes of the past and present. Contrib Mus Paleontol Univ Mich 13: 181-210.

S329. Webb LJ (1959) A physiognomic classification of Australian rain forests. J Ecol 47: 551-570.

S330. Ryder VL (1954) On the morphology of leaves. Bot Rev 20: 263-276.

S331. MacGinitie HD (1953) Fossil plants of the Florissant beds, Colorado. Carnegie Inst Washington Publ 599: 1-198.

S332. Richards, P. W. (1952) The tropical rain forest. Cambridge: Cambridge University Press. 450 p.

S333. Shields LM (1950) Leaf xeromorphy as related to physiological and structural influences. Bot Rev 16: 399-447.

S334. Dorf E (1942) Upper Cretaceous floras of the Rocky Mountain region II: flora of the Lance Formation at its type locality, Niobrara County, Wyoming. Carnegie Instiution of Washington Publication 508: 79-159.

S335. MacGinitie HD (1941) A middle Eocene flora from the central Sierra Nevada. Carnegie Inst Washington Publ 534: 1-178.

S336. Dorf E (1938) Upper Cretaceous floras of the Rocky Mountain region I: stratigraphy and palaeontology of the Fox Hills and lower Medicine Bow formations of southern Wyoming and northwestern Colorado. Carnegie Inst Washington Publ 508: 1-78.

S337. MacGinitie HD (1937) The flora of the Weaverville beds of Trinity County, California. Carnegie Inst Washington Publ 465: 83-151.

S338. Melville R (1937) The accurate definition of leaf shapes by rectangular coordinates. Ann Bot n s 1: 673-679.

S339. Potbury SS (1935) The La Porte flora of Plumas County, California. Carnegie Inst Washington Publ 465: 29-81.

S340. Sanborn EI (1935) The Comstock flora of west central Oregon. Carnegie Inst Washington Publ 465: 1-28.

S341. Chaney RW, Sanborn EI (1933) The Goshen flora of west central Oregon. Carnegie Inst Washington Publ 439: 1-103.

S342. Bews JW (1927) Studies in the ecological evolution of the angiosperms . New Phytol 26: 1-21.

S343. Chaney RW (1924) Quantitative studies of the Bridge Creek flora. Am J Sci 5th Ser 7: 127-145.

S344. Chaney RW (1920) The flora of the Eagle Creek Formation. Contributions from Walker Museum 2: 115-181.

S345. Brown WH (1919) Vegetation of the Philippine Mountains. Bur Sci (Manila) Publ 13: 1-433.

S346. Hanson HC (1917) Leaf-structure as related to environment. Am J Bot 4: 533-560.

S347. Bailey IW, Sinnott EW (1916) The climatic distribution of certain types of angiosperm leaves. Am J Bot 3: 24-39.

S348. Berry EW (1916) The lower Eocene floras of southeastern North America. U S Geol Surv Prof Pap 91: 1-481.

S349. Bailey IW, Sinnott EW (1915) A botanical index of Cretaceous and Tertiary climates. Science 41: 831-834.

S350. Billings FH (1905) Precursory leaf serrations of *Ulmus*. Bot Gaz 40: 224-225.

S351. Brenner W (1902) Klima und Blatt bei der Gattung *Quercus*. Flora 90: 114-160.
